# Kaiso protects human umbilical vein endothelial cells against apoptosis by differentially regulating the expression of B-cell CLL/lymphoma 2 family members

**DOI:** 10.1038/s41598-017-07559-0

**Published:** 2017-08-02

**Authors:** Xiaodong Xue, Jian Zhang, Huai Lan, Yinli Xu, Huishan Wang

**Affiliations:** Department of Cardiovascular Surgery, General Hospital of Shenyang Military Area Command, No.83, Wenhua Road, Shenhe District, Shenyang City, Liaoning 110016 China

## Abstract

Endothelial cell injury can promote the development of various cardiovascular diseases, thus, fully understanding the mechanisms underlying the maintenance of vascular endothelial cell homoeostasis may help prevent and treat cardiovascular disease. Kaiso, a zinc finger and BTB domain containing transcription factor, is key to embryonic development and cancer, but how Kaiso interacts with vascular endothelium is not fully understood. We report that Kaiso has an anti-apoptotic function in human umbilical vein endothelial cells (HUVECs) and human microvascular endothelial cells (HMEC-1s). Overexpression of Kaiso significantly increased cell viability and inhibited hydrogen peroxide-induced apoptosis. Furthermore, Kaiso increased expression of B-cell CLL/lymphoma 2 (BCL2) and reduced expression of BCL2-associated X protein (BAX) and BCL2-interacting killer (BIK) by differentially regulating gene promoter activity. Methylated DNA and specific Kaiso binding site (KBS) contributed to gene regulatory activity of Kaiso. In addition, p120ctn functioned cooperatively in Kaiso-mediated transcriptional regulation.

## Introduction

Vascular endothelial cells maintain homoeostasis of the vascular system by modulating vascular tone, platelet aggregation, inflammation, fibrinolysis, and proliferation of smooth muscle cells^1, 2^. Endothelial cell injury can promote the development of various cardiovascular diseases and evidence suggests that oxidative stress is the primary deleterious factor responsible for the impairment of endothelial cell function^1, 3–5^. Excessive production of reactive oxygen species (ROS), decreased nitric oxide, antioxidant system impairment, and an imbalance of vasoactive substances alter the redox state and signal transduction in endothelial cells, ultimately leading to mitochondrial dysfunction and apoptosis^5–7^. Multiple redox-sensitive signaling pathways and transcription factors reportedly participate in the oxidative stress response^8–11^, but mechanism underlying oxidative stress mediated vascular endothelial cell dysfunction is not fully understood.

Kaiso belongs to the BTB/POZ (broad complex, tramtrack, bric à brac/pox virus and zinc finger) family of zinc finger transcription factors^12, 13^. As a bi-modal DNA-binding transcription factor, Kaiso binds to methylated CpG dinucleotides and also to a sequence-specific Kaiso binding site (KBS),TCCTGCNA (where N represents any nucleotide), within target gene promoters through its zinc-finger (ZF) motif in the C-terminal region^14–16^. In addition, methylated CpG dinucleotides and KBS cooperate in Kaiso-mediated transcriptional regulation^17^. Recently, an orphan palindromic sequence, TCTCGCGAGA, was reported to be a DNA binding motif of Kaiso^18, 19^, in which the methylated CGCG core and the flanking sequences are important for Kaiso binding^19^. In addition, the relative Kaiso binding affinity of KBS is lower than the methylated palindromic site and much higher than the methylated CGCG core^19^. Upon DNA binding, Kaiso recruits a transcriptional co-repressor, such as nuclear receptor co-repressor (N-CoR), via its POZ domain in the N-terminal region to mediate transcriptional repression^15, 19, 20^. Kaiso can also function as a cofactor. For example, Kaiso heterodimerizes with another POZ-ZF member, Znf131, via its POZ domain to inhibit Znf131 mediated transcriptional repression in epithelial and fibroblast cells^21^.

It has been reported that Kaiso has a role in embryonic development and cancer. Kaiso is a negative regulator of canonical Wnt signaling, which is fundamental for embryonic development and tumor progression^22^. The depletion of xKaiso, a *Xenopus* homologue, was found to trigger apoptosis in early stage embryos and lead to gastrulation defects^23^. In the Apc^Min/+^ mouse model of intestinal cancer, Kaiso expression was upregulated in the intestinal cancer tissue and the absence of Kaiso extended lifespan and attenuated intestinal neoplasia^24^. On the contrary, in Apc^Min/+^ mice over-expressing Kaiso (Kaiso^Tg/+^:Apc^Min/+^), lifespan was reduced and polyp multiplicity was increased compared to Apc^Min/+^ mice^25^. Kaiso expression has been found to be upregulated in several kinds of human cancer, and cytoplasmic Kaiso expression has been associated with greater malignancy and poor prognoses^26–29^. In colon cancer cells, Kaiso contributes to the DNA methylation-dependent silencing of tumor suppressor genes^30^. However, recently Koh’s group found that Kaiso enhances apoptosis in human HEK293 and MEF cells in a p53-dependent manner. DNA damage induces Kaiso, which then interacts with p53-p300 complex via its POZ and ZF domains. This interaction increases the acetylation of p53 K320 and K382 residues and decreases K381 acetylation, which leads to increased p53-to-DNA binding, followed by the transcription of various apoptotic genes^31, 32^. Thus, Kaiso may have different functions in different cellular or gene contexts.

P120 catenin (p120ctn) was first identified as a Kaiso binding partner in a yeast two-hybrid screen^33^. P120ctn belongs to the subfamily of Armadillo repeat-containing proteins. In the vascular endothelium, p120ctn is well known for stabilizing cell-cell adhesion through regulating the turnover of VE-cadherin^34, 35^. In addition, p120ctn can translocate to the nucleus under thrombin stimulation in HUVECs, indicating a gene regulatory function for p120ctn^36^. Indeed, Kaiso and p120ctn have been reported to work cooperatively to regulate of gene transcription in cancer and endothelial cells^37, 38^.

However, the role of Kaiso in the vascular endothelium is unclear. Previous work suggests that Kaiso is abundantly expressed in several endothelial cell types, including bovine pulmonary artery endothelial cells (BPAECs); human microvascular endothelial cells from the lung, brain and dermis; and human coronary artery endothelial cells (HCAECs)^37, 39^. Also, Kaiso and p120ctn expression increased in cells at the wound border of an injured HCAEC monolayer to enhance wound closure^39^, indicating a protective effect of Kaiso in wounded endothelial cells. We report a role for Kaiso in oxidative stress-mediated vascular endothelial cell dysfunction. We found that Kaiso inhibited apoptosis induced by oxidative stress in HUVECs and HMEC-1s. Furthermore, Kaiso upregulated B-cell CLL/lymphoma 2 (BCL2) gene expression while down regulating the expression of BCL2-associated X protein (BAX) and BCL2-interacting killer (BIK) by differentially regulating promoter activity of these genes. Both methylated DNA and the specific Kaiso binding site (KBS) played roles in the gene regulatory activity of Kaiso. Moreover, p120ctn functioned cooperatively in the above Kaiso-mediated transcriptional regulation.

## Results

### Kaiso expression in H_2_O_2_ treated endothelial cells

**Figure 1:**
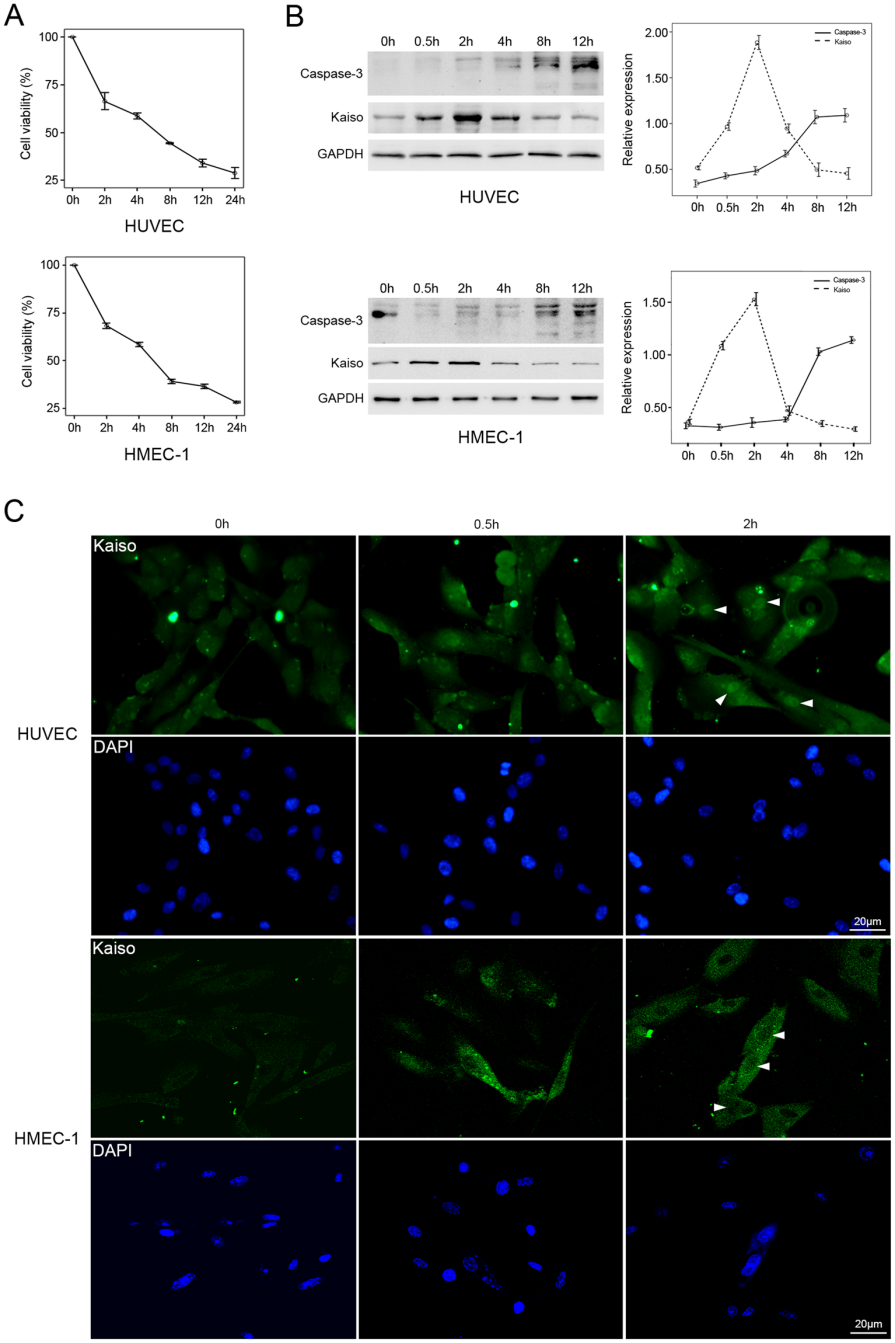
Kaiso expression in H_2_O_2_-treated endothelial cells. Cells were treated with 400 μM H_2_O_2_ for 0, 2, 4, 8, 12, and 24 h and cell viability was analyzed at the end of each time period using CCK8 method (**A**). Results are presented as mean ± SD of triplicate (n = 3) experiments. Cells were treated with 400 μM H_2_O_2_ for 0, 0.5, 2, 4, 8, and 12 h and the protein expressions of Kaiso and Caspase-3 were analyzed using Western blot (**B**, left panel). GAPDH served as loading control. Representative curves of relative protein expressions of Kaiso and Caspase-3 are presented as densitometric values corrected by GAPDH (**B**, right panel). Experiment was performed in triplicate (n = 3). Cells were treated with 400 μM H_2_O_2_ for 0, 0.5, and 2 h and stained with monoclonal mouse anti-Kaiso 6F/6F8-CHIP grade antibody (**C**). Cell nuclei were stained with DAPI. Arrowheads show the nuclear expression of Kaiso. Scale bar represents 20 μm.

As previously reported, 400 μM H_2_O_2_ effectively induces apoptosis in HUVEC^40^. HUVECs and HMEC-1s were treated with 400 μM H_2_O_2_ and assessed for cell viability. Data show that cell viability was significantly decreased after treatment with H_2_O_2_ for 8 h (Fig. 1A). Western blot confirmed increased Caspase-3 expression in both cell types after 8 h treatment with H_2_O_2_ (Fig. 1B) and documented apoptosis. H_2_O_2_ treatment upregulated Kaiso expression which peaked at 2 h post-treatment and decreased approximately 8 h post-treatment, a time at which significant increase in Caspase-3 expression occurred (Fig. 1B). Additionally, immunofluorescent staining showed that the nuclear expression of Kaiso increased from 0.5 to 2 h after H_2_O_2_ treatment in HUVECs and HMEC-1s groups (Fig. 1C, arrowheads). Thus, H_2_O_2_ treatment increased protein expression and the nuclear localization of Kaiso in HUVECs and HMEC-1s but in HMEC-1s cytoplasmic expression of Kaiso was significantly increased after H_2_O_2_ treatment for 2 h (Fig. 1C and Supplementary Figure S1), indicating that cytoplasmic Kaiso responds to oxidative stress in HMEC-1s. Oxidative stress can trigger rapid and tightly controlled changes in gene expression in mammalian cells and transcription, mRNA turnover and translation play major roles in this process^41^. The rapid upregulation of Kaiso expression induced by H_2_O_2_ treatment may be part of the oxidant-triggered gene expression program in endothelial cells. However, sustained exposure to toxic H_2_O_2_ can overwhelm cellular antioxidant function and induce apoptosis, and Kaiso mRNA and protein can be rapidly degraded once apoptosis is initiated.

### Selective silencing and overexpression of Kaiso in endothelial cells

**Figure 2:**
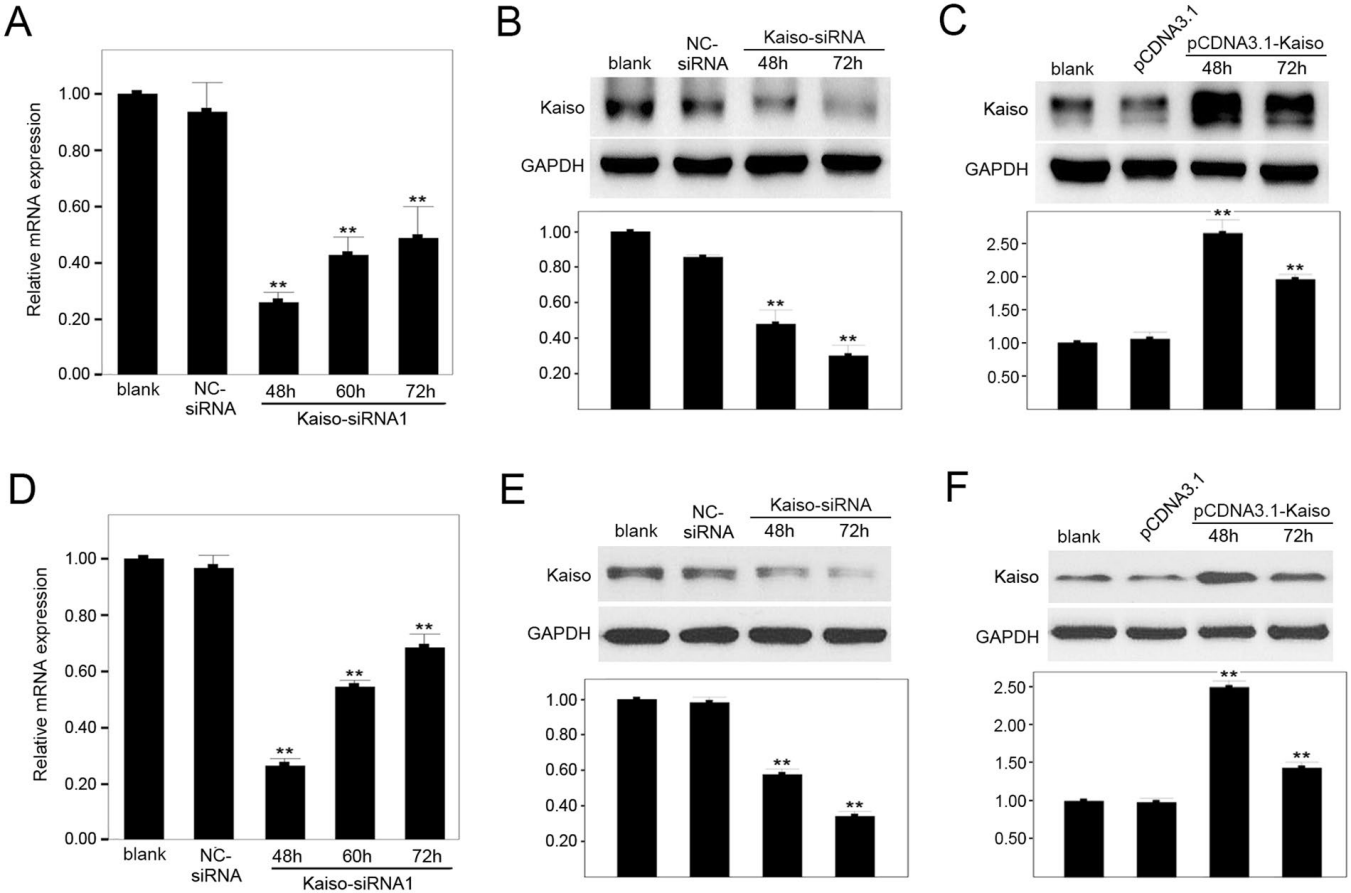
Selective silencing and overexpression of Kaiso in endothelial cells. HUVECs (**A**,**B**) and HMEC-1s (**D**,**E**) were transfected with NC-siRNA or Kaiso-siRNA1 and silencing efficiency was evaluated by quantitative real-time PCR (**A** and **D**) and Western blot (**B** and **E**) at 48, 60 or 72 h post transfection. HUVECs (**C**) and HMEC-1s (**F**) were transfected with pCDNA3.1 or pCDNA3.1-Kaiso and Kaiso expression was evaluated by Western blot at 48 and 72 h after transfection. GAPDH served as a loading control. Values are presented as mean ± SD, **p < 0.01, compared with blank, n = 3.

Short interfering RNA (siRNA) targeting homologous human Kaiso mRNA (Kaiso-siRNA1 and Kaiso-siRNA2) were separately transfected into HUVECs and HMEC-1s to silence Kaiso expression. Silencing efficiency was evaluated using quantitative real-time PCR and Western blot. It was found that, compared to blank (wild type, no treatment) or negative controls (NC-siRNA), Kaiso mRNA was significantly reduced 48, 60, and 72 h after Kaiso-siRNA transfection. The least mRNA was at 48 h after transfection (Fig. 2A and D and Supplementary Figure S2). Western blot showed that, compared with the blank or NC-siRNA group, Kaiso protein was significantly downregulated after Kaiso-siRNA transfection, with the least protein appearing at 72 h (Fig. 2B and E). This delay in reduced protein may be due to turnover of remnant products of Kaiso protein. To obtain Kaiso over-expression, pCDNA3.1-Kaiso was transfected into HUVECs and HMEC-1s. Western blot (Fig. 2C and F) show that Kaiso expression was increased at 48 and 72 h after transfection, and peaked at 48 h after transfection.

### Anti-apoptotic effect of Kaiso in H_2_O_2_ treated endothelial cells

**Figure 3:**
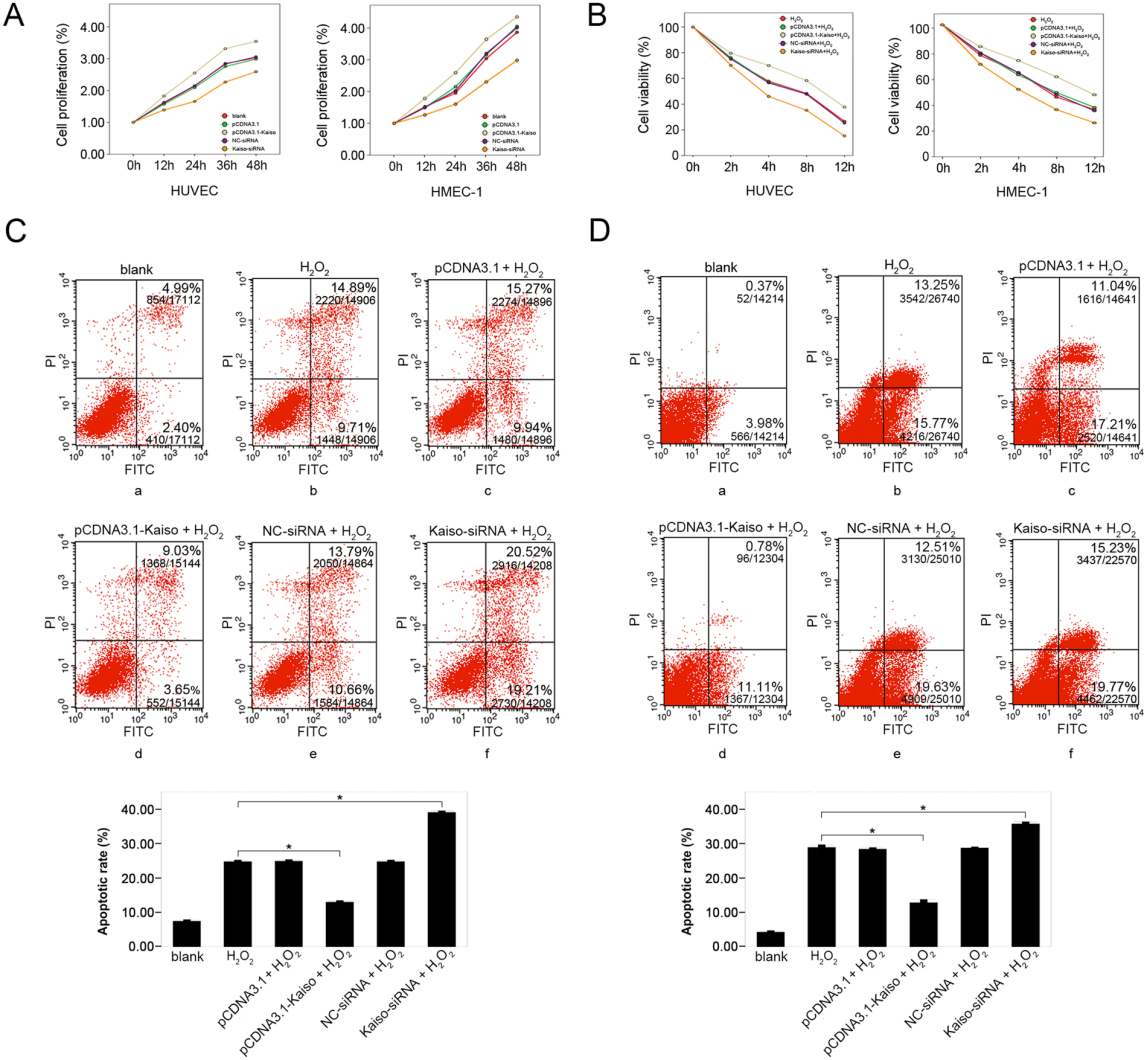
Anti-apoptotic effect of Kaiso in H_2_O_2_ treated endothelial cells. Cells were transfected with pCDNA3.1, pCDNA3.1-Kaiso, NC-siRNA or Kaiso-siRNA and cell viability was analyzed at 0, 12, 24, 36, and 48 h after transfection using CCK8 method (**A**). Cells were transfected with pCDNA3.1, pCDNA3.1-Kaiso, NC-siRNA or Kaiso-siRNA and were cultured at 37 °C for 48 h. Then, cells were treated with 400 μM H_2_O_2_ for 0, 2, 4, 8, and 12 h and cell viability was analyzed at the end of each time period using CCK8 method (**B**). HUVECs (**C**) and HMEC-1s (**D**) were transfected with pCDNA3.1, pCDNA3.1-Kaiso, NC-siRNA or Kaiso-siRNA and were cultured at 37 °C for 48 h. Cells were then treated with 400 μM H_2_O_2_ for 8 h and cell apoptosis was examined using Annexin V/Propidium iodide (PI) flowcytometry analysis. The untreated cells served as a blank. Values are presented as mean ± SD, *p < 0.01, compared with H_2_O_2_ group, n = 3.

To investigate the role of Kaiso in endothelial cells, wild type (blank), Kaiso knockdown (Kaiso-siRNA), Kaiso over-expressing (pCDNA3.1-Kaiso) and negative control (NC-siRNA and pCDNA3.1) cells were cultured for 0, 12, 24, 36, and 48 hours and cell proliferation was analyzed using the CCK8 method. In HUVECs, cell proliferation increased in the pCDNA3.1-Kaiso group and reduced in the Kaiso-siRNA group, compared to blank and negative controls (p < 0.01, n = 3) (Fig. 3A, left panel). Similar results were obtained in HMEC-1s (Fig. 3A, right panel). Thus, Kaiso promotes cell proliferation in endothelial cells. Cells from the above groups were also treated with 400 μM H_2_O_2_ for 0, 2, 4, 8, and 12 hours. CCK8 analysis show that, for HUVECs, cell viability was significantly increased in the pCDNA3.1-Kaiso + H_2_O_2_ group and reduced in the Kaiso-siRNA + H_2_O_2_ group compared with the H_2_O_2_ and negative control groups (pCDNA3.1 + H_2_O_2_ and NC-siRNA + H_2_O_2_) (p < 0.01, n = 3) (Fig. 3B, left panel). Similarly, for HMEC-1s, cell viability in the pCDNA3.1-Kaiso + H_2_O_2_ group was greater than in the H_2_O_2_ group, and viability in the Kaiso-siRNA + H_2_O_2_ group was less (Fig. 3B, right panel). In addition, an Annexin V/PI apoptosis assay performed 8 h after 400 μM H_2_O_2_ treatment show that, in HUVECs, about 25% cell apoptosis was induced in the H_2_O_2_ and negative control groups (pCDNA3.1 + H_2_O_2_ and NC-siRNA + H_2_O_2_) (Fig. 3Cb,c and e). Apoptosis was reduced in the pCDNA3.1-Kaiso + H_2_O_2_ group by 11.75 ± 0.45% (p < 0.01, n = 3) (Fig. 3Cd) and increased in the Kaiso-siRNA + H_2_O_2_ group by 14.33 ± 0.51%, compared with the H_2_O_2_ group (p < 0.01, n = 3) (Fig. 3Cf). Similarly, in HMEC-1s, apoptosis was reduced in the pCDNA3.1-Kaiso + H_2_O_2_ group by 16.41 ± 0.38% (p < 0.01, n = 3) (Fig. 3Dd) and increased in the Kaiso-siRNA + H_2_O_2_ group by 6.72 ± 1.3%, compared with the H_2_O_2_ group (p < 0.01, n = 3) (Fig. 3Df). These results demonstrate that Kaiso plays an anti-apoptotic role in H_2_O_2_ treated endothelial cells.

### Expression of BCL2, BAX and BIK in Kaiso genetically modified endothelial cells

**Figure 4:**
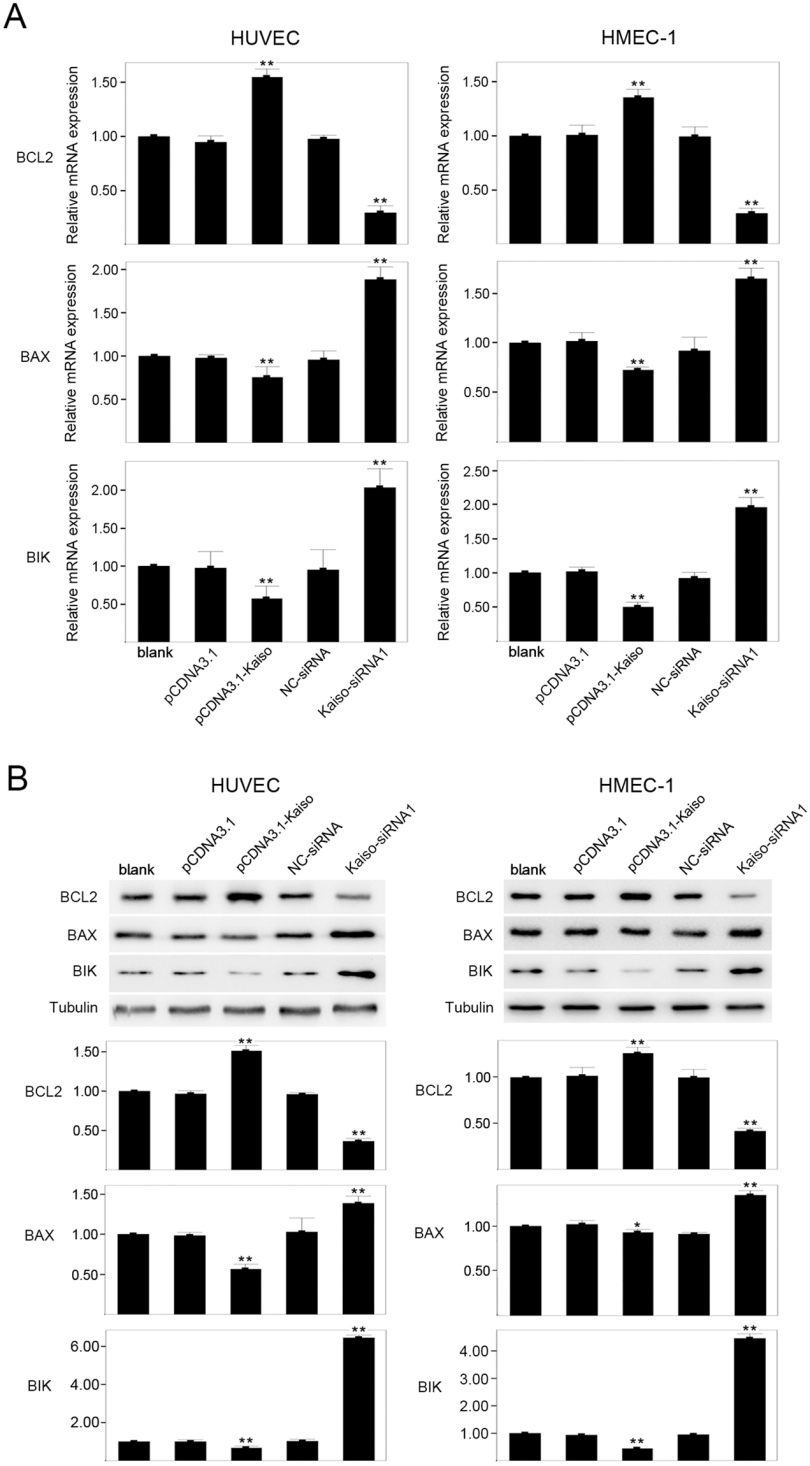
Expression of BCL2, BAX and BIK in Kaiso-modified endothelial cells. Cells were transfected with pCDNA3.1, pCDNA3.1-Kaiso, NC-siRNA or Kaiso-siRNA1 and were cultured at 37 °C for 48 h. The expression of BCL2, BAX and BIK was evaluated using quantitative real-time PCR (**A**) and Western blot (**B**). The untreated cells served as a blank. Tubulin served as a loading control. Values are presented as mean ± SD, **p < 0.01, *p < 0.05, compared with blank, n = 3.

BCL2, BAX and BIK belong to different subfamilies (BH1-4, BH1-3, and BH3, respectively) of the BCL2 protein family and all are important regulators of cell apoptosis. BCL2 inhibits cell apoptosis, whereas BAX and BIK promote cell apoptosis. We hypothesized that BCL2, BAX, and BIK could be target genes of Kaiso and that the anti-apoptotic effect of Kaiso, at least in part, could be due to the regulation of BCL2, BAX, and BIK expression by Kaiso. Expressions of BCL2, BAX, and BIK in HUVECs and HMEC-1s were analyzed, via quantitative real-time PCR and Western blot, in each of the following groups: blank, pCDNA3.1, pCDNA3.1-Kaiso, NC-siRNA and Kaiso-siRNA (Fig. 4 and Supplementary Figure S3). In HUVECs and HMEC-1s, Kaiso overexpression upregulated BCL2 mRNA and downregulated BAX and BIK mRNAs compared with blank (p < 0.01). In contrast, inhibition of Kaiso expression significantly downregulated BCL2 mRNA and upregulated the mRNAs of BAX and BIK (p < 0.01). Similarly, Western blot show that, in HUVECs and HMEC-1s, Kaiso overexpression upregulated BCL2 protein (p < 0.01) and downregulated BAX (p < 0.05) and BIK protein (p < 0.01), compared with blank. Conversely, Kaiso knockdown significantly decreased BCL2 protein and increased BAX and BIK protein expression (p < 0.01). HUVECs were co-transfected with pCDNA3.1-Kaiso and BCL2 siRNA as indicated and then treated with 400 μM H_2_O_2_ for 8 h. Expression of Kaiso, BCL2 and cleaved-Caspase-3 were analyzed by Western blot. Data show that Kaiso-mediated inhibition of Caspase-3 activation was compromised by BCL2 knockdown in HUVECs (Supplementary Figure S4). These results suggest that the anti-apoptotic effect of Kaiso in endothelial cells could be at least partly due to the regulation of BCL2, BAX, and BIK transcription.

### Kaiso binds to BCL2, BAX, and BIK promoters

**Figure 5:**
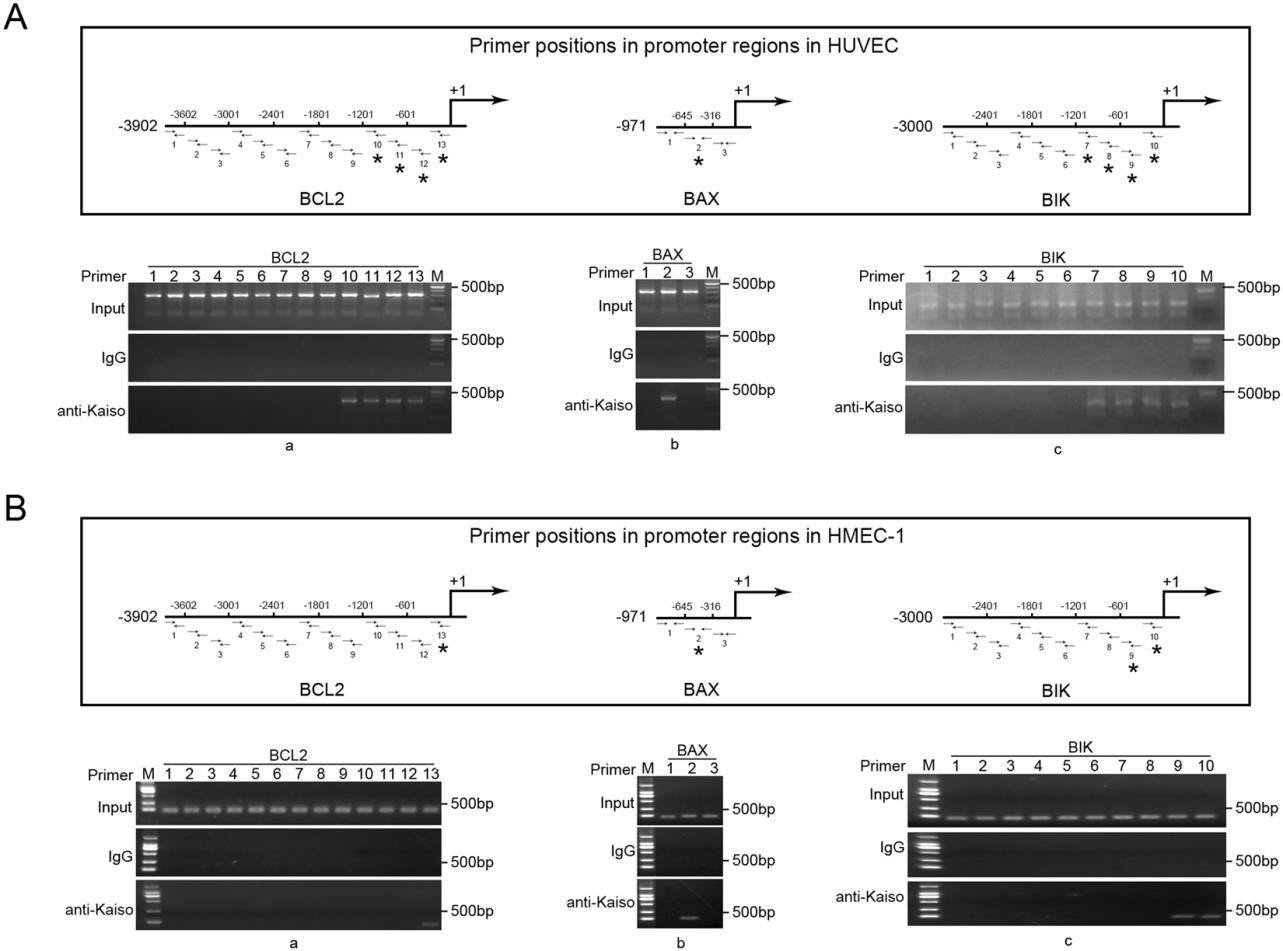
Kaiso binds to the promoters of BCL2, BAX, and BIK. DNA fragments (200–500 bp) were obtained from nuclear lysates of HUVECs (**A**) and HMEC-1s (**B**), respectively, and were subjected to chromatin immunoprecipitation. Mouse monoclonal anti-Kaiso 6F/6F8-CHIP grade antibody was used for Kaiso/DNA complex precipitation and the precipitated DNA fragments were amplified using primers targeting promoter regions of BCL2 (−3902 to −1 bp) (**A**,a and **B**,a), BAX (−971 to −1 bp) (**A**,b and **B**,b) and BIK (−3000 to −1 bp) (**A**,c and **B**,c). Primer positions (horizontal arrows) are shown in the schematic drawings in the upper panel in A and B. Asterisks mark the positively amplified promoter regions. Normal mouse IgG was used as a negative control. M: DNA marker. (**A**) In HUVECs, Kaiso binds to the promoter regions −1201 to −1 bp of BCL2, −645 to −316 bp of BAX and −1201 to −1 bp of BIK (Asterisks). (**B**) In HMEC-1s, Kaiso binds to the promoter regions −301 to −1 bp of BCL2, −645 to −316 bp of BAX and −601 to −1 bp of BIK (Asterisks). Detailed primer positions and sequences were listed in Supplementary Table S2.

To confirm that Kaiso regulates the expression of BCL2, BAX, and BIK through binding to the promoter regions of these genes, chromatin immunoprecipitation (CHIP) was performed in untreated, wild type HUVECs and HMEC-1s. Kaiso antibody and primers directed against the promoter regions of BCL2 (−3902 to −1 bp, GenBank: EU119400.1), BAX (−971 to −1 bp, GenBank: AB183034.1) and BIK (3000 bps immediately upstream to the open reading frame, NT_011520, 24498–27498 bp) were used (Fig. 5, schematic drawings, Supplementary Table S2). The BCL2 promoter region of −1201 to −1 bp was amplified using primer 10–13 in HUVECs (Fig. 5Aa), though only region −301 to −1 bp was amplified using primer 13 in HMEC-1s (Fig. 5Ba). PCR products were obtained for BAX using primer 2, which corresponded to the promoter region −645 to −317 bp in HUVECs and HMEC-1s (Fig. 5Ab and B,b). For BIK, the amplified PCR products were obtained using primers 7–10, which corresponds to the promoter region −1200 to −1 bp in HUVECs (Fig. 5Ac), though only −601 to −1 bp were amplified using primers 9 and 10 in HMEC-1s (Fig. 5Bc). These results demonstrate that Kaiso specifically binds to the promoter regions of BCL2, BAX, and BIK, indicating that Kaiso has a regulatory function in gene expression.

### Kaiso binds to BCL2 and BAX promoters, at least in part, via methylated DNA

**Figure 6:**
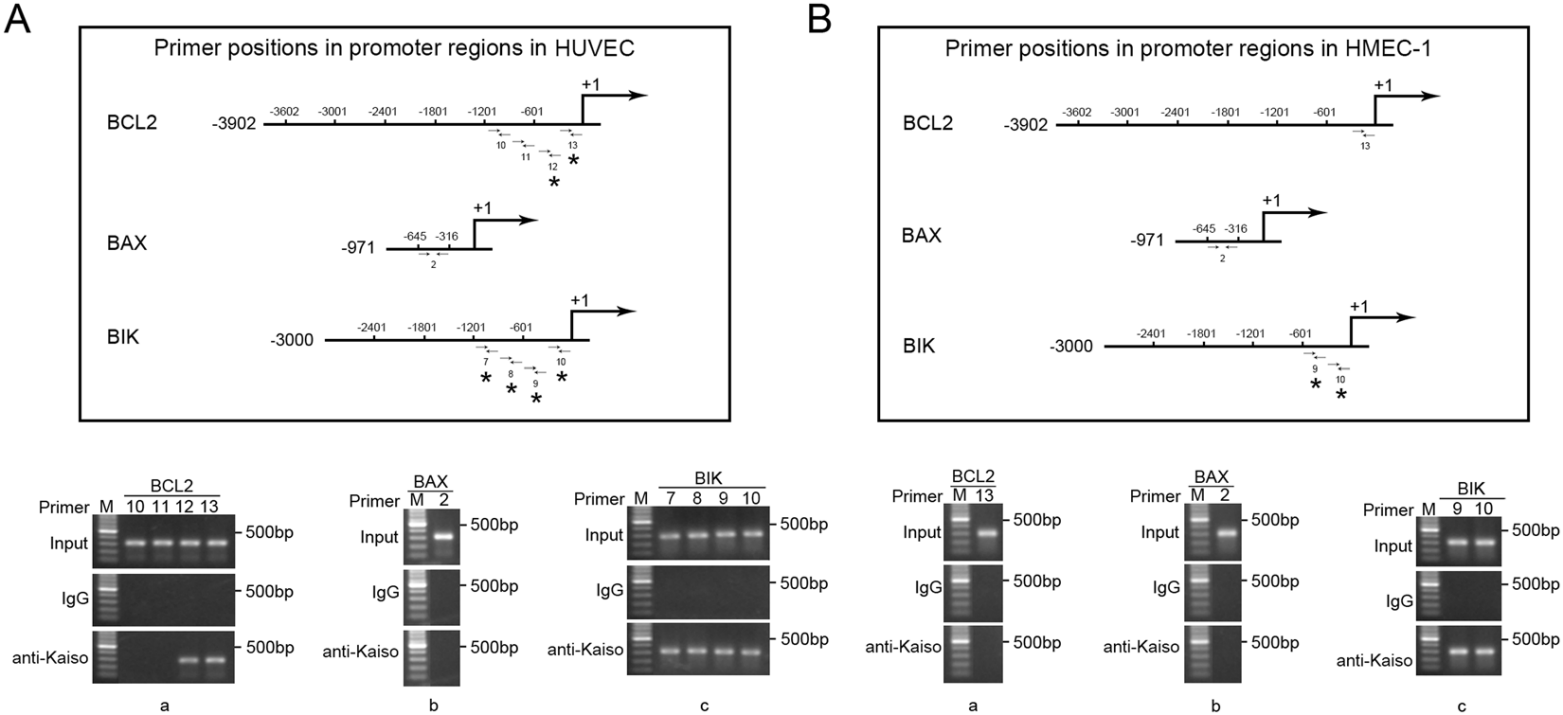
Kaiso binds to the promoters of BCL2 and BAX, at least partly, via methylated DNA. HUVECs (**A**) and HMEC-1s (**B**) were treated with 5 μM 5-azacytidine for 72 h. DNA fragments (200–500 bp) were obtained from nuclear lysates and were subjected to chromatin immunoprecipitation. Mouse monoclonal anti-Kaiso 6F/6F8-CHIP grade antibody was used for Kaiso/DNA complex precipitation and the precipitated DNA fragments were amplified using primers 10–13 (BCL2) (**A**,a and **B**,a), primer 2 (BAX) (**A**,b and **B**,b) and primer 7–10 (BIK) (**A**,c and **B**,c). Primer positions (horizontal arrows) are shown in the schematic drawings in the upper panel in A and B. Asterisks mark the positively amplified promoter regions. Normal mouse IgG was used as a negative control. M: DNA marker. (**A**) In HUVECs, after 5-azacytidine mediated DNA demethylation, Kaiso remained binding to the promoter regions −601 to −1 bp of BCL2 and −1201 to −1 bp of BIK (Asterisks) while no longer binding to the promoter region −645 to −316 bp of BAX. (**B**) In HMEC-1s, after 5-azacytidine mediated DNA demethylation, Kaiso remained binding to the promoter region −601 to −1 bp of BIK (Asterisks) while no longer binding to the promoter regions −1201 to −1 bp of BCL2 and −645 to −316 bp of BAX. DNA methylation/demethylation in BAX promoter region −661 to −301 bp was determined using methylation specific PCR (Supplementary Figure S5).

Kaiso is a methylated DNA binding transcription factor, thus the role of DNA methylation in the promoter binding activity of Kaiso was evaluated. Cells were treated with 5 μM 5-azacytidine for 72 h to achieve DNA demethylation (Supplementary Figure S5) and then subjected to CHIP assay using the primers positively guided DNA amplification in the previous CHIP (Fig. 5). Data show that after 5-azacytidine treatment, primers targeting the BCL2 promoter region −1200 to −601 bp in HUVECs (Fig. 6Aa) and −301 to −1 bp in HMEC-1s (Fig. 6Ba) and the BAX promoter region −645 to −316 bp in both HUVEC (Fig. 6Ab) and HMEC-1 (Fig. 6Bb) failed to guide DNA fragment amplification. Thus, Kaiso binds to BCL2 and BAX promoters via DNA methylation, at least in part. The other primers successfully guided DNA amplification after 5-azacytidine treatment (Fig. 6, *in schematic drawings), indicating a role for KBS or the unmethylated palindromic site, TCTCGCGAGA, in Kaiso-to-promoter binding. However, sequence analysis confirmed no TCTCGCGAGA site in promoters of BCL2, BAX, and BIK, suggesting the functionality of KBS in Kaiso-to-promoter binding.

### KBSs take part in Kaiso-mediated transcriptional regulation of BCL2, BAX and BIK

**Figure 7:**
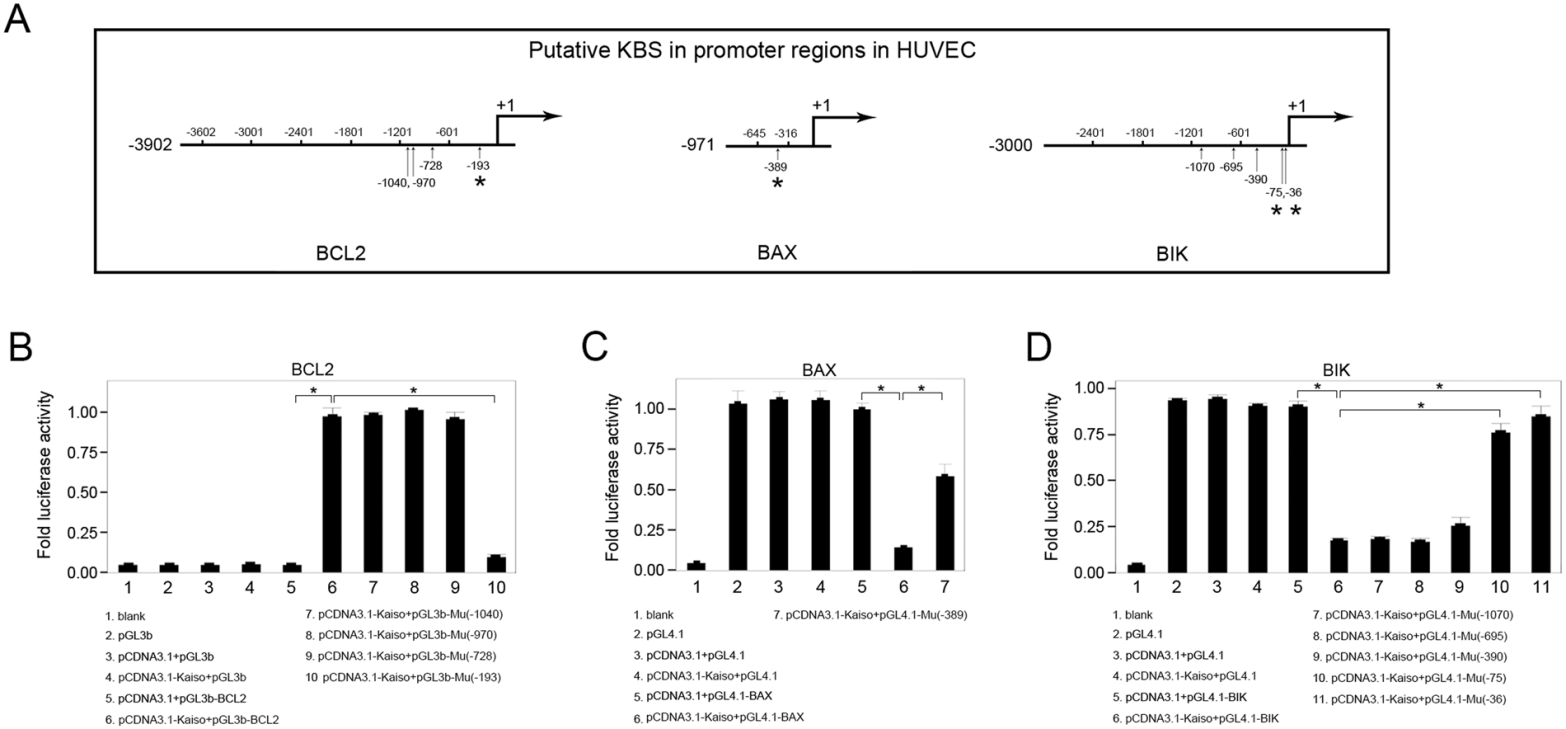
KBSs take part in the Kaiso mediated transcriptional regulation of BCL2, BAX and BIK. HUVECs were transfected with recombinant firefly luciferase reporter constructs pGL3b-BCL2 (**B**), pGL4.1-BAX (**C**) or pGL4.1-BIK (**D**) together with pCDNA3.1 or pCDNA3.1-Kaiso. The KBS-mutated constructs were negative controls. *Renilla* luciferase reporter pRL-TK was an internal control. The untreated cells served as a blank. Relative luciferase activity is presented as mean ± SD. *p < 0.01, n = 3. Positions of putative KBSs (vertical arrows) are shown in **A**. Asterisks in A mark the KBS mutations that mediated reversion of Kaiso regulatory effects. (**B**–**D**) Mutations of KBS (−193) in BCL2 promoter, KBS (−389) in BAX promoter and KBS (−75, −36) in BIK promoter (asterisks in **A**) significantly reversed Kaiso mediated transcriptional regulation. Site mutations in putative KBSs were listed in Supplementary Table S5.

Promoters of BCL2, BAX, and BIK were analyzed to study the role of KBS in the transcription regulatory activity of Kaiso and 4, 1, and 5 putative KBSs, respectively, were predicted from BCL2 promoter, BAX promoter and BIK promoter (Fig. 7A). Genomic sequences corresponding to these promoter regions were subcloned into pGL3-Basic or pGL4.1, to generate firefly luciferase reporter constructs. Additionally, each putative KBS in the reporter constructs was selectively mutated using site-directed mutagenesis (Supplementary Table S5). HUVECs were cotransfected with the firefly luciferase reporter constructs and the *Renilla* luciferase reporter vector pRL-TK together with pCDNA3.1 or pCDNA3.1-Kaiso. Luciferase reporter assay data show that, the relative luciferase activity in the pCDNA3.1-Kaiso + pGL3b-BCL2 group was significantly higher than in the pCDNA3.1 + pGL3b-BCL2 group (0.973 ± 0.02 vs 0.05 ± 0.0007, p < 0.01, n = 3) (Fig. 7B). In contrast, the relative luciferase activity in the pCDNA3.1-Kaiso + pGL4.1-BAX group was significantly lower than in the pCDNA3.1 + pGL4.1-BAX group (0.14 ± 0.003 vs 1.0 ± 0.02, p < 0.01, n = 3) (Fig. 7C). Similarly, relative luciferase activity in the pCDNA3.1-Kaiso + pGL4.1-BIK group was lower than in the pCDNA3.1 + pGL4.1-BIK group (0.17 ± 0.005 vs 0.9 ± 0.01, p < 0.01, n = 3) (Fig. 7D). Thus, Kaiso enhances promoter activity of BCL2 while repressing promoter activity of BAX and BIK. These promoter regulatory effects of Kaiso were significantly reversed by selective KBS mutations as relative luciferase activity in the pCDNA3.1-Kaiso + pGL3b-Mu (−193) group was significantly lower than in the pCDNA3.1-Kaiso + pGL3b-BCL2 group (0.09 ± 0.007 vs 0.97 ± 0.02, p < 0.01, n = 3) (Fig. 7B). In contrast, relative luciferase activity in the pCDNA3.1-Kaiso + pGL4.1-Mu (−389) group was significantly higher than in the pCDNA3.1-Kaiso + pGL4.1-BAX group (0.58 ± 0.03 vs 0.14 ± 0.003, p < 0.01, n = 3) (Fig. 7C). In addition, relative luciferase activity in the pCDNA3.1-Kaiso + pGL4.1-Mu(−75) and pCDNA3.1-Kaiso + pGL4.1-Mu(−36) groups was significantly higher than in the pCDNA3.1-Kaiso + pGL4.1-BIK group (0.76 ± 0.02 and 0.85 ± 0.02 vs 0.17 ± 0.005, p < 0.01, n = 3) (Fig. 7D). Thus, Kaiso regulates promoter activity of BCL2, BAX and BIK, at least in part, by binding with KBSs at these promoter positions.

**Figure 8:**
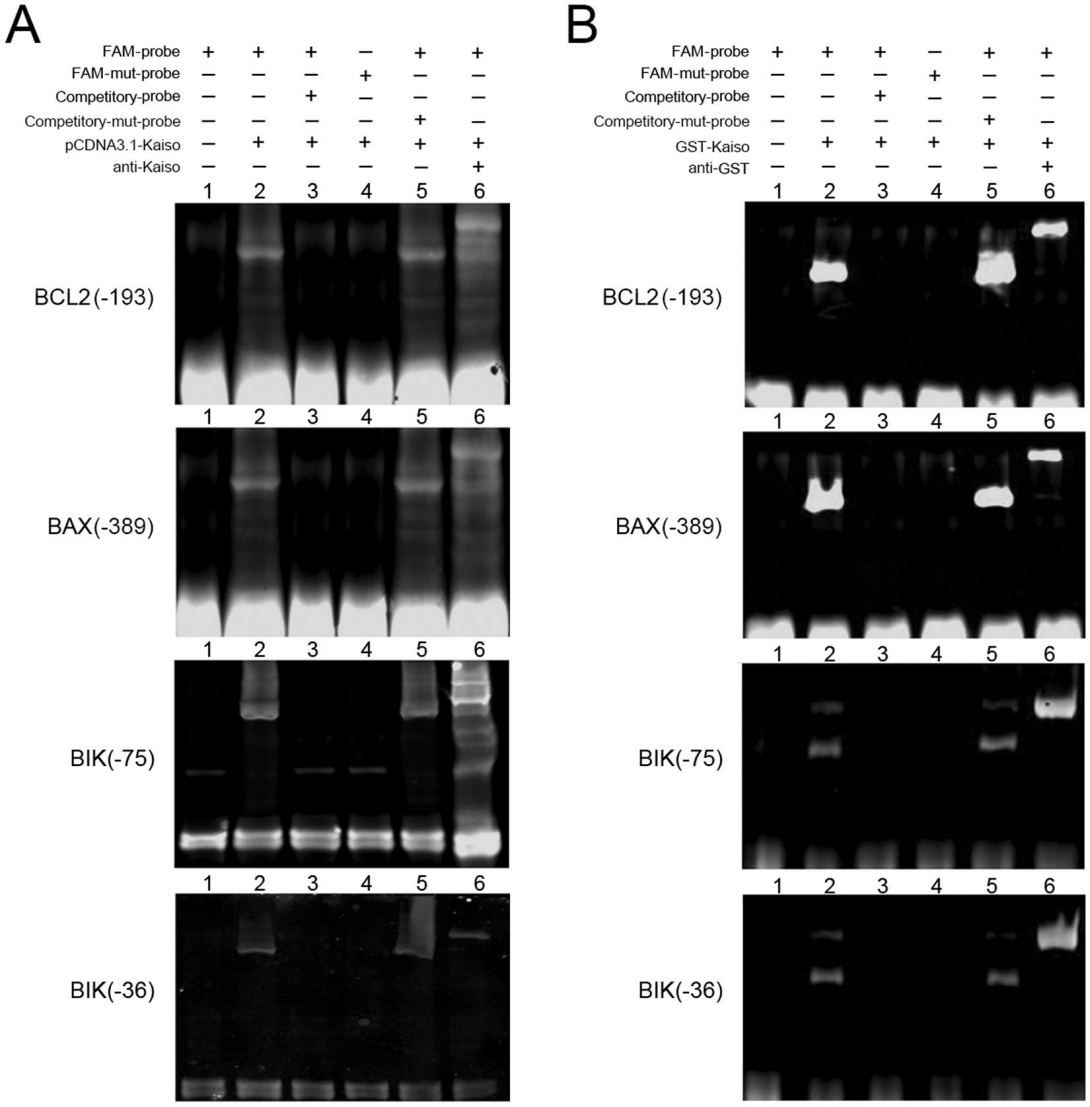
Kaiso binds to specific KBSs on BCL2, BAX and BIK promoter. Nonradioactive EMSA was performed using probes targeted to the promoter positions that contain putative KBSs (BCL2: −193 bp, BAX: −389 bp, BIK: −75 bp, −36 bp). Wild type probes were labeled with 6-FAM and were incubated with cell nuclear extracts from HUVECs overexpressing Kaiso (**A**, lane 2–5) or with recombinant protein GST-Kaiso (**B**, lane 2–5). Labeled wild type probes (FAM-probe) alone served as a negative control (lane 1). Monoclonal mouse anti-Kaiso 6F/ 6F8-CHIP grade antibody (**A**, lane 6) and monoclonal mouse anti-GST antibody (**B**, lane 6) were used to detect super gel shift. Unlabeled wild type probes (Competitory-probe) served as competition. KBS-mutated probes (FAM-mut-probe and Competitory-mut-probe) served as a negative control and competition.

A nonradioactive electrophoretic mobility shift assay (EMSA) was performed to confirm Kaiso-KBS binding at the above promoter positions. Probes targeting promoter positions, which contained putative KBSs (BCL2: −193 bp, BAX: −389 bp, BIK: −75 bp, −36 bp) were labeled with 6-FAM and then incubated with cell nuclear extracts. Incubated mixtures were resolved with SDS-PAGE and the fluorescent signals were recorded using a fluorescent scanner. Gel shifts were observed when labeled probes were incubated with nuclear extracts from HUVECs over-expressing Kaiso (Fig. 8A, lane 2). Gel shifts disappeared when competitory non-labeling probes were added to the incubations (Fig. 8A, lane 3), suggesting binding of Kaiso to labeled probes. No gel shift was observed when the putative KBSs in the labeled probes were mutated (Fig. 8A, lane 4). In addition, KBS-mutated probes did not disturb the gel shifts generated by labeled probes (Fig. 8A, lane 5). This indicated binding of Kaiso to the putative KBS. Super gel shifts were observed when the Kaiso antibody and the labeled probes were incubated with the nuclear extracts from HUVECs over-expressing Kaiso (Fig. 8A, lane 6), which demonstrated the presence of Kaiso protein in the gel shifting complexes. Similar results were obtained when EMSA was performed again using recombinant protein, GST-Kaiso, instead of cell nuclear extracts (Fig. 8B). These results confirm the direct binding of Kaiso to the above specific KBSs in BCL2, BAX, and BIK promoters.

### P120ctn participates in BCL2, BAX and BIK gene regulation by Kaiso

**Figure 9:**
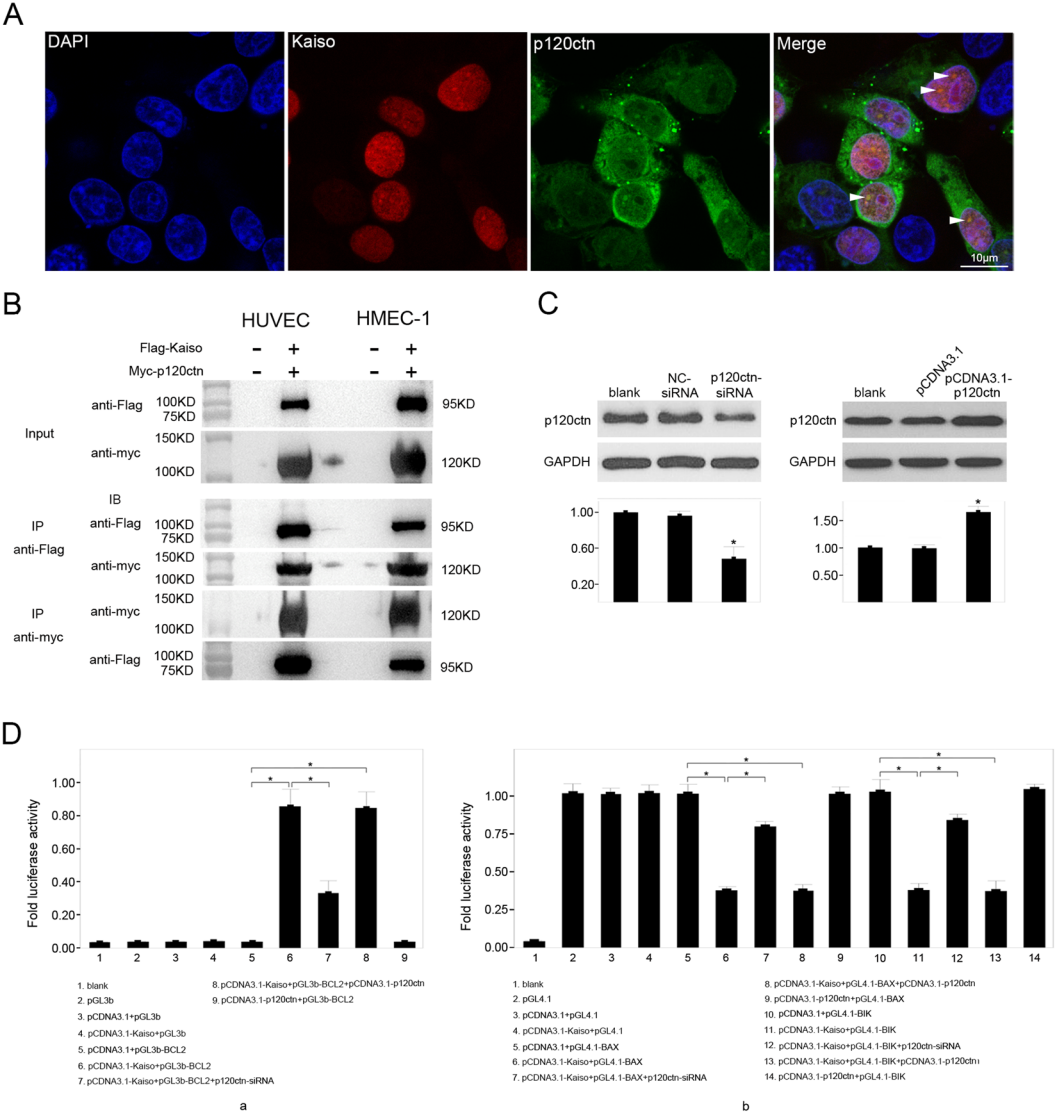
P120ctn participates in the gene regulation of BCL2, BAX and BIK by Kaiso. HUVECs were transfected with pCDNA3.1-Kaiso for 48 h and stained with polyclonal rabbit anti-Kaiso (H-154) antibody (in red color) and monoclonal mouse anti-p120ctn antibody (in green color) (**A**). Cell nuclei were stained with DAPI (**A**,a). Arrowheads show the particle like structures where both Kaiso and p120ctn were observed (**A**,d). Scale bar represents 10 μm. HUVECs and HMEC-1s were co-transfected with pCMV-flag-Kaiso and pCMV-myc-p120ctn, and 48 h later the interaction of Kaiso and p120ctn was evaluated by co-immunoprecipitation using monoclonal mouse anti-FLAG antibody and monoclonal mouse anti-Myc tag antibody respectively (**B**). HUVECs were transfected with NC-siRNA, p120ctn-siRNA, pCDNA3.1 and pCDNA3.1-p120ctn respectively, and p120ctn expression was evaluated by Western blot 48 h after transfection (**C**). GAPDH served as a loading control. Values are presented as mean ± SD, *p < 0.01, compared with blank, n = 3. HUVECs were transfected with recombinant firefly luciferase reporter constructs pGL3b-BCL2 (**D**,a), pGL4.1-BAX and pGL4.1-BIK (**D**,b), together with pCDNA3.1, pCDNA3.1-Kaiso, pCDNA3.1-Kaiso and p120ctn-siRNA, pCDNA3.1-Kaiso and pCDNA3.1-p120ctn, and pCDNA3.1-p120ctn, respectively. *Renilla* luciferase reporter pRL-TK was used as an internal control. The untreated HUVECs served as a blank. Relative luciferase activity is presented as mean ± SD. *p < 0.01, n = 3.

As a binding partner of Kaiso, p120ctn was speculated to participate in BCL2, BAX, and BIK gene regulation by Kaiso. Subcellular distribution of Kaiso and p120ctn upon Kaiso overexpression in HUVECs was examined using immunofluorescent double staining (Fig. 9A) and data show overexpressed Kaiso protein was mainly located in nuclei and a large proportion of p120ctn protein was located in cytoplasm. Similar to the previous observation in MDCK and NIH3T3 cells^33^, Kaiso and p120ctn were both located on several particle-like structures in the nuclear matrix (Fig. 9A, arrowheads), which could be composed of heterochromatin, as was previously reported in mouse embryonic stem cells^44^. Therefore, Kaiso and p120ctn may be functionally correlated in endothelial cells.

To determine whether p120ctn works as a binding partner of Kaiso in endothelial cells, co-immunoprecipitation was performed using HUVECs and HMEC-1s over-expressing Flag-Kaiso and Myc-p120ctn (Fig. 9B). In HUVECs and HMEC-1s, Flag-Kaiso and Myc-p120ctn mutually precipitated each other using anti-Flag and anti-Myc antibody, respectively. This result demonstrates that Kaiso and p120ctn can bind to each other in endothelial cells.

To further study the role of p120ctn in the transcriptional regulatory function of Kaiso, siRNA targeting human p120ctn mRNA and pCDNA3.1-p120ctn, respectively, were transfected into HUVECs and p120ctn protein level was significantly downregulated or upregulated compared to controls 48 h after transfection (Fig. 9C). Luciferase reporter assay data confirmed that knockdown of p120ctn expression significantly reversed promoter regulatory effects of Kaiso (Fig. 9D). Relative luciferase activity in the pCDNA3.1-Kaiso + pGL3b-BCL2 + p120ctn-siRNA group was significantly lower than in the pCDNA3.1-Kaiso + pGL3b-BCL2 group (Fig. 9Da). In contrast, relative luciferase activity for pCDNA3.1-Kaiso + pGL3b-BAX + p120ctn-siRNA and pCDNA3.1-Kaiso + pGL3b-BIK + p120ctn-siRNA groups were significantly higher than in the pCDNA3.1-Kaiso + pGL3b-BAX and pCDNA3.1-Kaiso + pGL3b-BIK groups (Fig. 9Db). However, p120ctn overexpression did not affect promoter transcriptional activity for the genes studied (Fig. 9D). Thus, p120ctn can function cooperatively with Kaiso in BCL2, BAX and BIK gene regulation in endothelial cells.

## Discussion

Kaiso is a member of the BTB-POZ transcription factor family, although the exact function of Kaiso in the vascular endothelium is unclear. We present evidence that Kaiso plays an anti-apoptotic role in endothelial cells.

ROS are the main damaging factors that cause vascular endothelial cell injury. In this study, we noted that Kaiso expression was rapidly upregulated after H_2_O_2_ treatment, strongly suggesting the involvement of Kaiso during oxidative stress in endothelial cells. Additionally, our data show that Kaiso overexpression increased cell viability and protected endothelial cells from H_2_O_2_-induced apoptosis. Thus, it appears that Kaiso is a protective factor that helps maintain homeostasis of the vascular endothelium. However, these data contrast with those by Koh’s group who reported that Kaiso overexpression promoted apoptosis in human HEK293 and MEF cells^31, 32^. They found that ectopic Kaiso changed the acetylation manner of p53, resulting in upregulation of p53 target genes. This paradox could be due to the different cell lines that were used in their research.

Several lines of evidence indicate that Kaiso is a transcriptional repressor in various cell types, including endothelial cells^22, 30, 37, 38, 45^. Kaiso recruits nuclear receptor co-repressor (N-CoR) via its POZ domain to promote repressive chromatin structure formation in the target gene promoter and repress transcription^15^. In contrast, Kaiso was reported to be a transcriptional activator regulating the expression of rapsyn, a synapse-specific protein, in C2C12 myocytes and at the neuromuscular junction^46^. We report that Kaiso can function as a transcriptional repressor and an activator. Kaiso inhibits transcription of BAX and BIK, while activating transcription of BCL2, thus rebalancing pro- and anti- apoptotic gene expression and leading to inhibition of apoptosis. This is similar to the POZ-ZF member, ZF5, which is related to Kaiso. ZF5 has been reported to activate HIV-1 LTR and repress the β-actin promoter and its POZ domain is required for transcriptional activation and repression^13, 47, 48^. However, the mechanism that determines whether ZF5 acts as a transcriptional activator or a repressor is not fully understood. Of note, within transcription factors, domains rich in acidic amino acids are usually related to transcriptional activation. For example, transcriptional activator Miz-1 (ZBTB17) and ZF-5 contain highly acidic domains upstream of their zinc fingers. Similarly, in Kaiso, there are two highly acidic regions (aa187-aa195 and aa327-aa338), which are downstream to the POZ domain, indicating a transcriptional activation function.

Our CHIP data show that Kaiso binds to the promoter regions of BCL2 (−301 to −1 bp), BAX (−645 to −316 bp) and BIK (−601 to −1 bp) in HUVECs and HMEC-1s, suggesting the existence of Kaiso response elements in these regions. Because Kaiso is a methylated DNA binding transcription factor, methylated DNA may participate in Kaiso-to-promoter binding in HUVECs and HMEC-1s, at least in part. CHIP data show that the contribution of DNA methylation to Kaiso-to-promoter binding differs between the two cell types and among the three genes. In BCL2, DNA methylation appeared non-essential for Kaiso binding to the promoter region −301 to −1 bp in HUVECs. However, in HMEC-1s, DNA methylation was required for Kaiso to bind to the same region. In BAX, DNA methylation was necessary for Kaiso-to-promoter binding in both HUVECs and HMEC-1s. In contrast, Kaiso binding to the BIK promoter occurred irrespective of DNA methylation. These data indicate that, in addition to methylated DNA, other DNA binding motifs also participate in Kaiso-to-promoter binding. Given that no TCTCGCGAGA site exists in the BCL2, BAX, and BIK promoter, KBS may play roles in Kaiso-to-promoter binding.

Data from the luciferase reporter assay showed that intact specific KBSs are required for the Kaiso-mediated gene regulation of BCL2, BAX and BIK. As expected, specific KBSs in BCL2 (5′-TCCTGCAT-3′, −193 bp), BAX (5′-TCCTGCCT-3′, −389 bp) and BIK (5′-TCCTGTGA-3′, −75 bp and 5′-TCCAGTCA-3′, −36 bp) promoters mediate the transcriptional regulatory effects of Kaiso to different extents. The binding of Kaiso to specific KBSs was confirmed with EMSA. Among the KBSs, KBS (−193 bp) and KBSs (−75 bp and −36 bp) exhibited high efficiency in gene regulation while KBS (−389 bp) was less efficient. This is consistent with the CHIP data showing that methylated DNA is not essential for Kaiso-to-DNA binding in BCL2 and BIK promoters (except for the BCL2 promoter in HMEC-1s), indicating that KBS plays a major role in the regulation of these genes. On the other hand, the requirement for methylated DNA in Kaiso-to-promoter binding in BAX indicates that, other than KBS, DNA methylation may play a cooperative role in the repression of BAX transcription. This agrees with the work of Donaldson’s group who reported that methylated CpG dinucleotides helped to stabilize Kaiso’s interaction with KBS in the transcriptional repression of cyclinD1 in HCT116 and MCF7 cells^17^.

P120ctn is the major binding partner of Kaiso and has been reported to promote the dissociation of Kaiso from KBS, thus relieving Kaiso-mediated transcriptional repression^22, 42, 43^. However, our luciferase reporter assay data show that knockdown of p120ctn expression reversed the promoter regulatory effects of Kaiso in BCL2, BAX and BIK, which suggests cooperative activity of p120ctn in Kaiso-mediated gene regulation. This agrees with previously reported data that p120ctn acts collaboratively and efficiently with Kaiso in repressing KBS reporter activity in human brain microvascular endothelial cells (HBMECs), which depend on Kaiso-to-KBS binding^37^. Similarly, delta-catenin, a brain-specific member of the p120 catenin subfamily, served as a co-activator in Kaiso mediated transcription activation of rapsyn in mouse neuromuscular junction^46^. Nevertheless, the overexpression of p120ctn had no significant effect on the promoter activities of BCL2, BAX and BIK, or Kaiso mediated gene regulation. These data suggest that p120ctn functions as a collaborator of Kaiso in gene transcriptional regulation in endothelial cells, which may depend on Kaiso-to-DNA binding. As an abundantly expressed housekeeper protein, the endogenous p120ctn may be able to fully exert influence on the efficacy of Kaiso-to-DNA binding, which may not be significantly changed by ectopic p120ctn. Since p120ctn cooperates with Kaiso in transcriptional activation and repression, it is more likely that p120ctn plays a permissive role in Kaiso-mediated gene regulation, at least in part, via stabilization of Kaiso-to-DNA binding. Data from the current study on p120ctn is distinct from observations in other cell types. The mechanisms and reasons for this discrepancy are not yet understood. It is known that the p120ctn binding site in Kaiso is a noncontiguous epitope that flanks the ZF domain (aa348–453 and aa632–638), which mediates the inhibition of Kaiso-to-DNA binding^16^. Other than this noncontiguous epitope, the zinc finger 1 is another possible p120ctn binding site in Kaiso, which may facilitate regulation of Kaiso transcriptional properties by p120ctn, possibly via steric hindrance of Kaiso-to-DNA binding or inducing conformational change in Kaiso^16^. Thus in endothelial cells, the binding of p120ctn to zinc finger 1 may induce a conformational change in Kaiso that leads to a higher DNA binding affinity or a greater availability of a co-factor binding motif, like the POZ domain.

Moreover, the above promoter regulatory effects of Kaiso were only partially reversed by the knock down of p120ctn expression, suggesting that additional co-factors took part in the transcriptional regulatory function of Kaiso. It has been reported that Kaiso represses matrix metalloproteinase-7 (MMP7) expression in conjunction with transcriptional co-repressor myeloid translocation gene 16 (MTG16) through binding to the KBS in the MMP7 promoter^20^. Thus, MTG16 could be a candidate co-repressor of Kaiso in the regulation of BAX and BIK promoters. In addition, N-CoR could be a co-factor in Kaiso-mediated BAX promoter inhibition via DNA methylation. For Kaiso mediated BCL2 promoter activation, there is little information describing Kaiso as a transcriptional activator or any co-activator in Kaiso-mediated transcriptional regulation. The above mentioned transcriptional activation of rapsyn mediated by Kaiso and delta-catenin is myocyte specific, which indicates additional cell type specific co-factors are necessary in the transcriptional activation effect of Kaiso^46^. Recently, Koh and colleagues reported that Kaiso increased transcription of p53 target genes in human HEK293 cells and MEF cells^31, 32^. However, this effect depended on p53-to-p53 response element binding, not Kaiso-to-DNA binding. Instead, Kaiso served as a co-factor, which changed the acetylation manner of p53 resulting in a higher DNA binding affinity of p53. Nonetheless, as mentioned above, Kaiso contains two highly acidic regions downstream to the POZ domain, which might mediate the transcriptional activation of BCL2. In addition, the POZ domain is required in Kaiso-Kaiso homodimerization, Kaiso-Znf131 heterodimerization, Kaiso/N-CoR complex formation^15, 21, 33^ and in the transcriptional activation activity of ZF5^47^. Thus, Kaiso may mediate transcriptional activation by recruiting co-activators via the POZ domain.

Recently, a bioinformatic study performed by Blattler’s group showed that, instead of binding to methylated DNA and mediating transcriptional inhibition *in vitro*, Kaiso binds unmethylated regions of the genome *in vivo*, which is associated with transcriptional activation^18^. They reported that Kaiso mostly bound to active promoters that are highly marked with hypomethylation, H3K9 and H3K27 acetylation. They also excluded the possibility of Kaiso-to-methylated DNA binding in several cancer cell lines as the tightly packed methylated DNA restricts the access of transcription factors; however, they did not show any direct experimental evidence in individual genes. In addition, their study focused on Kaiso-to-methylated DNA binding without focusing much attention on Kaiso-to-KBS binding. Thus, based on their evidence, it is difficult to determine whether KBS plays a major role in Kaiso-mediated transcriptional activation *in vivo*. Nonetheless, the work of Blattler and colleagues indicates an even more complex mechanism behind Kaiso’s function.

In summary, these data suggest that Kaiso can inhibit endothelial apoptosis via differentially regulating expression of BCL2, BAX, and BIK and that methylated DNA and specific Kaiso binding site (KBS) contribute to the gene regulatory activity of Kaiso. As a binding partner of Kaiso, p120ctn functions cooperatively in the above transcriptional regulation mediated by Kaiso. These findings suggest that Kaiso may be a feasible target for developing new therapeutic strategies for treating cardiovascular disease.

## Methods

### Cells and reagents

Human umbilical vein endothelial cells (HUVECs) and human microvascular endothelial cells-1 (HMEC-1s) were purchased from American Type Culture Collection (ATCC, Rockefeller, MD) and maintained in Endothelial Cell Medium (ECM) (ScienCell, Grand Isle, NY) supplemented with 10% fetal bovine serum (FBS), 1% Glutamine, 100 U/ml penicillin, and 100 mg/ml streptomycin. Cells were treated with 400 μM PERDROGEN^™^ (H_2_O_2_) (Sigma Aldrich, Santa Clara, CA) in all the apoptosis induction experiments.

### RNA interference

Selective gene silencing was achieved using small interfering RNA (siRNA). Synthetic-siRNA targeting homologous human Kaiso-mRNA (Kaiso-siRNA1, sense: 5′-CAUAUGCCCUCUUCAAUCAtt-3′, antisense: 5′-UGAUUGAAGAGGGCAUAUGat-3′; Kaiso-siRNA2, sense: 5′-GGCCGUCAGUAAUACAUCUtt-3′, antisense: 5′-AGAUGUAUUACUGACGGCCga-3′) and human -p120ctn mRNA (p120ctn-siRNA, sense: 5′-GCUCGCAACAAAGAAUUAAtt-3′, antisense: 5′-UUAAUUCUUUGUUGCGAGCat-3′) for degradation were purchased from Life Technologies (Carlsbad, CA). BCL2 siRNA was purchased from Santa Cruz Biotechnology (Dallas, TX). Cell transfection was performed using Lipofectamine 2000 transfection reagent (Life Technologies). The day before transfection, HUVECs were seeded into a 12-well plate and cultured at 37 °C and 24 h later, medium was changed and siRNA of Kaiso (Kaiso-siRNA), p120ctn (p120ctn-siRNA), beta-Actin (NC-siRNA) (as a negative control) and GAPDH (GAPDH-siRNA) (as a positive control), respectively, were added to the fresh medium at a final concentration of 40 nM. Blank cells were treated with transfection reagent only. After transfection, cells were cultured at 37 °C for 24 to 72 h.

### Plasmid construction and cell transfection

The expression vectors pCDNA3.1-Kaiso, pCMV-flag-Kaiso, pCDNA3.1-p120ctn, and pCMV-myc-p120ctn were constructed by subcloning the cDNA of Kaiso (GenBank No: NM_001184742) and p120ctn (GenBank No: NM_001085458) in frame into pCDNA3.1 (Life Technologies), or pCMV-Tag2 and pCMV-Tag3 vectors (Agilent Tech, Santa Clara, CA), respectively, downstream to the human cytomegalovirus (CMV) promoter. Cells were seeded at a density of 5 × 10^4^ cells/ml in 24-well plates 24 h before transfection. Transfection was performed using Lipofectamine 2000 transfection reagent (Life Technologies) and the transfection conditions were optimized according to the manufacturer’s instructions. Briefly, for each well, 0.5 μg of plasmid DNA and 1 μl of Lipofectamine 2000 reagent were diluted separately in 25 μl Opti-MEM I reduced serum medium (Invitrogen, Carlsbad, CA) without serum. Dilutions were combined and incubated for 20 min, and then added into each well containing 0.5 ml culture medium without antibiotics. Finally, 8 h after transfection, medium was changed.

### Cell viability assay

Cell viability was analyzed using a Cell Counting Kit-8 (CCK-8) (Dojindo Molecular Technologies, Shanghai, China). Briefly, cells were incubated on 96-well plates (8,000 cells/well) and cultured for 24 h, then 10 μl of CCK-8 solution was added into each well and the plate was incubated for 2 h. OD was read at 450 nm on a microplate reader. Data are presented as means of three independent experiments.

### Western blot

Whole cell extracts were obtained by lysing cells in RIPA buffer (50 mM Tris-HCl (pH 7.4), 150 mM NaCl, 1% Triton X-100, 1% sodium deoxycholate, 0.1% SDS, 2 mM sodium pyrophosphate, 25 mM β-glycerophosphate, 1 mM EDTA, 1 mM Na_3_VO_4_, 0.5 μg/ml leupeptin). Nuclear and cytoplasmic extracts were prepared separately using NE-PER nuclear and cytoplasmic extraction reagents (Thermo-Scientific) according to kit instructions. Protein samples were resolved using 8% SDS-PAGE, transferred to PVDF membrane (EMD Millipore, Billerica, MA) and probed with the primary antibodies at 4 °C overnight, then washed and incubated with the secondary antibodies for 1 h at room temperature. Reactive bands were developed using SuperSignal West Pico chemiluminescent detection reagents (Thermo-Scientific, Rockford, IL). Antibodies used were monoclonal rabbit anti-Caspase-3 antibody and polyclonal rabbit anti-cleaved-Caspase-3 antibody (Cell Signaling Technology, Boston, MA), monoclonal mouse anti-Kaiso 6F/ 6F8-CHIP grade antibody (Abcam, Cambridge, UK), polyclonal rabbit anti-BCL2 antibody (Proteintech, Chicago, IL), polyclonal rabbit anti-BAX antibody (Proteintech), monoclonal mouse anti-BIK antibody (Cell Signaling Technology), monoclonal mouse anti-p120 Catenin antibody (BD Biosciences, San Jose, CA), monoclonal rabbit anti-GAPDH antibody (Cell Signaling Technology) (loading control), polyclonal rabbit anti-Histone H3 antibody (Proteintech) (loading control), horseradish peroxidase (HRP)-conjugated goat anti-mouse IgG (Proteintech) and HRP-conjugated goat anti-rabbit IgG (Proteintech).

### Quantitative real-time PCR

Total RNA was extracted using Trizol reagent (Life Technologies). First-strand cDNA was synthesized using the SuperScript III First-Strand Synthesis System (Life Technologies). Real-time PCR was performed using SYBR Green Real-Time PCR Master Mixes (Life Technologies). Primers used are listed in Supplementary Table S1.

### Immunofluorescence

Cells were seeded on coverslips and when they reached 80% confluence, they were rinsed with PBS and fixed in 4% paraformaldehyde for 20 min, and then washed two times in PBS and treated in 0.3% TritonX-100 for 10 min for membrane permeabilization. Cells were then blocked with 4% BSA in PBST for 1 h at room temperature and incubated with the primary antibodies at 4 °C overnight, then washed five times in PBS and incubated with the secondary antibodies for 1 h at room temperature. Stained cells were washed five times in PBS and mounted with ProLong Gold antifade reagent with DAPI (Invitrogen). Antibodies used were monoclonal mouse anti-Kaiso 6F/6F8-CHIP grade antibody (Abcam), polyclonal rabbit anti-Kaiso (H-154) antibody (Santa Cruz Biotechnology), monoclonal mouse anti-p120 Catenin antibody (BD Biosciences), phycoerythrin (PE)-conjugated goat anti-rabbit IgG and fluorescein isothiocyanate (FITC)-conjugated goat anti-mouse IgG (Jackson ImmunoResearch, West Grove, PA). Microscopic pictures were captured under a Zeiss LSM-710 confocal microscope (Zeiss, Oberkochen, Germany).

### Annexin V/Propidium iodide (PI) apoptosis assay

Annexin V/PI apoptosis assay was performed using Annexin V-FITC apoptosis detection kit I (BD Biosciences). Briefly, 1 × 10^6^ cells were collected and washed in PBS for two times, then resuspended in 500 μl binding buffer. Resuspended cells were incubated with Annexin V-FITC and PI for 15 min, and confirmed with a BD FASAria Cell Sorter (Beckton Dickinson, San Jose, CA).

### Chromatin immunoprecipitation (CHIP)

CHIP was performed in untreated wild type HUVECs and HMEC-1s using an EZ-Magna ChIP G Chromatin Immunoprecipitation Kit (Merck Millipore, Billerica, MA) according to the manufacturer’s instructions. Briefly, 1 × 10^7^ cells were fixed in formaldehyde at room temperature for 10 min. Fixation was terminated with glycine. Cells were lysed in cell lysis buffer, centrifuged and resuspended in nuclear lysis buffer. Nuclear lysates were sonicated on ice and were centrifuged at 12,000 × g at 4 °C for 10 min. Supernatants were subjected to agarose gel analysis and DNA fragments were confirmed to have average of 200–500 base pairs. The chromatin solution was diluted and incubated with antibodies and protein G magnetic beads at 4 °C overnight. Incubated protein G beads were washed. Protein/DNA complexes were eluted with ChIP Elution Buffer and Protein/DNA cross-link was reversed by adding proteinase K and incubating at 62 °C for 2 h with shaking. DNA was purified using spin columns and then subjected to PCR amplification using primers directed against the promoter regions of BCL2 (GenBank: EU119400.1), BAX (GenBank: AB183034.1), and BIK (NT_011520, 24498–27498 bp). For DNA demethylation, cells were treated with 5 μM 5-azacytidine for 72 h, and CHIP was performed again using primers positively guided DNA amplification in the previous assay. The used antibodies are: monoclonal mouse anti-Kaiso 6F/ 6F8-CHIP grade (Abcam) and normal mouse IgG (negative control). All primers used are shown in Supplementary Table S2.

### Methylation Specific PCR (MSP)

Genomic DNA was extracted from treated or untreated HUVECs and HMEC-1s with 5-azacytidine using DNeasy Blood & Tissue Kit (Qiagen, Hilden, Germany) and DNA bisulfite conversion was performed using an EpiTect Plus DNA Bisulfite kit (Qiagen) according to the manufacture’s guide. Converted DNA was detected by PCR using methylated and unmethylated primer sets (Supplementary Table S3). PCR was performed under the condition of pre-denaturation for 3 min at 94 °C followed by 35 cycles of 94 °C, 30 sec, 55 °C, 30 sec and 72 °C, 30 sec. PCR products were resolved on 3% agarose gel (Supplementary Figure S5).

### Luciferase Reporter Assay

According to the results of CHIP, four, one and five putative KBSs, respectively, were predicted from the promoter regions of human BCL2 (positions −1201 to −1), BAX (positions −645 to −317) and BIK (positions −1200 to −1). The above promoter regions were amplified from genomic DNA and were subcloned into firefly luciferase reporter vector pGL3-Basic (pGL3b) or pGL4.1 (Promega, Madison, WI) to get pGL3b-BCL2, pGL4.1-BAX and pGL4.1-BIK. Constructs with mutations of the putative KBSs were generated using mutagenic oligonucleotide primers according to the manual of the GeneTailor Site-Directed Mutagenesis System (Invitrogen). HUVECs were cotransfected with firefly luciferase reporter constructs and *Renilla* luciferase reporter vector pRL-TK (E2241, Promega) together with pCDNA3.1-Kaiso or pCDNA3.1 empty vector using FuGENE HD Transfection Reagent (Roche, Mannheim, Germany). Then, 48 h after transfection, relative luciferase activity represented as the ratio of firefly to *Renilla* was measured with a Dual-Luciferase Reporter Assay System (Promega). All primers used for genomic DNA amplification and site-directed mutation are listed in Supplementary Tables S4–S5.

### Electrophoretic Mobility Shift Assay (EMSA)

Nonradioactive electrophoretic mobility shift assay (EMSA) was performed. Briefly, 6-carboxylfluorescein (6-FAM) labeled oligonucleotides targeting to the promoter regions of BCL2, BAX, and BIK were synthesized (Sangon Biotech, Shanghai, China) and annealed. Labeled wild type probes (FAM-probe) alone served as a negative control. Unlabeled wild type probes (competitory probe) were competition. KBS-mutated probes (FAM-mut-probe and competitive-mut-probe) were negative controls and competition. Probes were incubated with either nuclear extracts from Kaiso over-expressing cells or with recombinant protein, GST-Kaiso (Abnova, Taipei, Taiwan). Accordingly, monoclonal mouse anti-Kaiso 6F/ 6F8-CHIP grade antibody (Abcam) or monoclonal mouse anti-GST antibody (Abnova), respectively, were added into the probe/nuclear extracts mixtures to generate a super shift. After incubation, mixtures were resolved with SDS-PAGE. Gels were photographed with a LI-COR Odyssey fluorescence scanner (LI-COR Biotech, Lincoln, NE). All probes used are listed in Supplementary Table S6.

### Co-Immunoprecipitation

Cells were co-transfected with pCMV-flag-Kaiso and pCMV-myc-p120ctn and 48 h after transfection, coimmunoprecipitation was performed using Pierce Co-IP kit (Thermo-Scientific). Briefly, cells were lysed in ice-cold IP Lysis Buffer. Cell lysate was pre-cleared by incubating with control agarose resin and spin down through a 1× coupling buffer-treated column. Then, pre-cleared cell lysate was incubated with antibody-coupled resin at 4 °C overnight in spin columns. After incubation, columns were washed in wash buffer and the bait:prey protein complex was eluted in elution buffer. The eluted protein complexes were examined using Western blot. Antibodies used were monoclonal mouse anti-FLAG antibody (Sigma Aldrich), monoclonal mouse anti-Myc tag antibody (Sigma Aldrich), HRP-conjugated goat anti-mouse IgG (Proteintech).

### Statistical Analysis

Data were analyzed using SPSS version 19.0 for Windows (SPSS, Chicago, IL). All values are presented as mean ± standard deviation (SD). One-way ANOVA with Tukey’s post hoc test was used to compare numeric data among the experimental groups (p < 0.05 was considered statistically significant).

## Electronic supplementary material

Supplementary Materials

## References

[1] Higashi Y, Noma K, Yoshizumi M, Kihara Y (2009). Endothelial function and oxidative stress in cardiovascular diseases. Circulation journal: official journal of the Japanese Circulation Society.

[2] Madoiwa S (2015). Recent advances in disseminated intravascular coagulation: endothelial cells and fibrinolysis in sepsis-induced DIC. Journal of intensive care.

[3] Libby P (2001). Current concepts of the pathogenesis of the acute coronary syndromes. Circulation.

[4] Lee HS (2004). Hydrogen peroxide-induced alterations of tight junction proteins in bovine brain microvascular endothelial cells. Microvasc Res.

[5] Li H, Horke S, Forstermann U (2014). Vascular oxidative stress, nitric oxide and atherosclerosis. Atherosclerosis.

[6] Deanfield JE, Halcox JP, Rabelink TJ (2007). Endothelial function and dysfunction: testing and clinical relevance. Circulation.

[7] Forstermann U (2008). Oxidative stress in vascular disease: causes, defense mechanisms and potential therapies. Nature clinical practice. Cardiovascular medicine.

[8] Chen B, Lu Y, Chen Y, Cheng J (2015). The role of Nrf2 in oxidative stress-induced endothelial injuries. The Journal of endocrinology.

[9] Tang Y, Jacobi A, Vater C, Zou X, Stiehler M (2014). Salvianolic acid B protects human endothelial progenitor cells against oxidative stress-mediated dysfunction by modulating Akt/mTOR/4EBP1, p38 MAPK/ATF2, and ERK1/2 signaling pathways. Biochemical pharmacology.

[10] Li A (2015). Arctigenin suppresses transforming growth factor-beta1-induced expression of monocyte chemoattractant protein-1 and the subsequent epithelial-mesenchymal transition through reactive oxygen species-dependent ERK/NF-kappaB signaling pathway in renal tubular epithelial cells. Free radical research.

[11] Yan S (2015). Clematichinenoside inhibits VCAM-1 and ICAM-1 expression in TNF-alpha-treated endothelial cells via NADPH oxidase-dependent IkappaB kinase/NF-kappaB pathway. Free radical biology & medicine.

[12] Albagli O, Dhordain P, Deweindt C, Lecocq G, Leprince D (1995). The BTB/POZ domain: a new protein-protein interaction motif common to DNA- and actin-binding proteins. Cell growth & differentiation: the molecular biology journal of the American Association for Cancer Research.

[13] Bardwell VJ, Treisman R (1994). The POZ domain: a conserved protein-protein interaction motif. Genes Dev.

[14] Prokhortchouk A (2001). The p120 catenin partner Kaiso is a DNA methylation-dependent transcriptional repressor. Genes Dev.

[15] Yoon HG, Chan DW, Reynolds AB, Qin J, Wong J (2003). N-CoR mediates DNA methylation-dependent repression through a methyl CpG binding protein Kaiso. Mol Cell.

[16] Daniel JM, Spring CM, Crawford HC, Reynolds AB, Baig A (2002). The p120(ctn)-binding partner Kaiso is a bi-modal DNA-binding protein that recognizes both a sequence-specific consensus and methylated CpG dinucleotides. Nucleic Acids Res.

[17] Donaldson NS (2012). Kaiso represses the cell cycle gene cyclin D1 via sequence-specific and methyl-CpG-dependent mechanisms. PLoS One.

[18] Blattler A (2013). ZBTB33 binds unmethylated regions of the genome associated with actively expressed genes. Epigenetics & chromatin.

[19] Raghav SK (2012). Integrative genomics identifies the corepressor SMRT as a gatekeeper of adipogenesis through the transcription factors C/EBPbeta and KAISO. Mol Cell.

[20] Barrett CW (2012). Kaiso directs the transcriptional corepressor MTG16 to the Kaiso binding site in target promoters. PLoS One.

[21] Donaldson NS (2010). Kaiso regulates Znf131-mediated transcriptional activation. Exp Cell Res.

[22] Park JI (2005). Kaiso/p120-catenin and TCF/beta-catenin complexes coordinately regulate canonical Wnt gene targets. Dev Cell.

[23] Ruzov A (2004). Kaiso is a genome-wide repressor of transcription that is essential for amphibian development. Development.

[24] Prokhortchouk A (2006). Kaiso-deficient mice show resistance to intestinal cancer. Mol Cell Biol.

[25] Pierre CC (2015). Kaiso overexpression promotes intestinal inflammation and potentiates intestinal tumorigenesis in Apc(Min/+) mice. Biochim Biophys Acta.

[26] Soubry A (2005). Expression and nuclear location of the transcriptional repressor Kaiso is regulated by the tumor microenvironment. Cancer Res.

[27] Dai SD (2009). Cytoplasmic Kaiso is associated with poor prognosis in non-small cell lung cancer. BMC Cancer.

[28] Dai SD (2010). Kaiso is expressed in lung cancer: its expression and localization is affected by p120ctn. Lung Cancer.

[29] Dai SD (2011). Upregulation of delta-catenin is associated with poor prognosis and enhances transcriptional activity through Kaiso in non-small-cell lung cancer. Cancer Sci.

[30] Lopes EC (2008). Kaiso contributes to DNA methylation-dependent silencing of tumor suppressor genes in colon cancer cell lines. Cancer Res.

[31] Koh DI (2014). KAISO, a critical regulator of p53-mediated transcription of CDKN1A and apoptotic genes. Proc Natl Acad Sci USA.

[32] Koh DI (2015). Transcriptional activation of APAF1 by KAISO (ZBTB33) and p53 is attenuated by RelA/p65. Biochim Biophys Acta.

[33] Daniel JM, Reynolds AB (1999). The catenin p120(ctn) interacts with Kaiso, a novel BTB/POZ domain zinc finger transcription factor. Mol Cell Biol.

[34] Davis MA, Ireton RC, Reynolds AB (2003). A core function for p120-catenin in cadherin turnover. J Cell Biol.

[35] Xiao K (2003). Cellular levels of p120 catenin function as a set point for cadherin expression levels in microvascular endothelial cells. J Cell Biol.

[36] Beckers CM, Garcia-Vallejo JJ, van Hinsbergh VW, van Nieuw Amerongen GP (2008). Nuclear targeting of beta-catenin and p120ctn during thrombin-induced endothelial barrier dysfunction. Cardiovasc Res.

[37] Zhang, J. *et al*. P120 catenin represses transcriptional activity through Kaiso in endothelial cells. *Microvasc Res* **80**, 233–239, S0026-2862(10)00081-6 [pii] doi:10.1016/j.mvr.2010.04.001 (2010). 10.1016/j.mvr.2010.04.001 PMC2917640 20382170

[38] Liu Y (2014). Kaiso interacts with p120-catenin to regulate beta-catenin expression at the transcriptional level. PLoS One.

[39] Kondapalli J, Flozak AS, Albuquerque ML (2004). Laminar shear stress differentially modulates gene expression of p120 catenin, Kaiso transcription factor, and vascular endothelial cadherin in human coronary artery endothelial cells. J Biol Chem.

[40] Hou X (2015). Dihydromyricetin protects endothelial cells from hydrogen peroxide-induced oxidative stress damage by regulating mitochondrial pathways. Life sciences.

[41] Abdelmohsen K, Kuwano Y, Kim HH, Gorospe M (2008). Posttranscriptional gene regulation by RNA-binding proteins during oxidative stress: implications for cellular senescence. Biological chemistry.

[42] Kim SW (2004). Non-canonical Wnt signals are modulated by the Kaiso transcriptional repressor and p120-catenin. Nat Cell Biol.

[43] Kelly KF, Spring CM, Otchere AA, Daniel JM (2004). NLS-dependent nuclear localization of p120ctn is necessary to relieve Kaiso-mediated transcriptional repression. J Cell Sci.

[44] Lehnertz B (2003). Suv39h-mediated histone H3 lysine 9 methylation directs DNA methylation to major satellite repeats at pericentric heterochromatin. Current biology: CB.

[45] Pierre CC (2015). Methylation-dependent regulation of hypoxia inducible factor-1 alpha gene expression by the transcription factor Kaiso. Biochim Biophys Acta.

[46] Rodova M, Kelly KF, VanSaun M, Daniel JM, Werle MJ (2004). Regulation of the rapsyn promoter by kaiso and delta-catenin. Mol Cell Biol.

[47] Kaplan J, Calame K (1997). The ZiN/POZ domain of ZF5 is required for both transcriptional activation and repression. Nucleic Acids Res.

[48] Numoto M (1993). Transcriptional repressor ZF5 identifies a new conserved domain in zinc finger proteins. Nucleic Acids Res.

