# Supplementary material for: Kaiso protects human umbilical vein endothelial cells against apoptosis by differentially regulating the expression of B-cell CLL/lymphoma 2 family members
Source: Sci Rep. 2017 Aug 2;7:7116. doi: 10.1038/s41598-017-07559-0 (PMC5540925; doi:10.1038/s41598-017-07559-0)
Supplement: Supplementary file 1 — Supplementary Materials [file 41598_2017_7559_MOESM1_ESM.pdf]

Kaiso protects human umbilical vein endothelial cell against apoptosis by differentially regulating the expressions of B-cell CLL/lymphoma 2 family members

Xiaodong Xue[1], Jian Zhang[1], Huai Lan[1], Yinli Xu[1] and Huishan Wang[1*]

[1]Department of Cardiovascular Surgery, General Hospital of Shenyang Military Area Command, No.83, Wenhua Road, Shenhe District, Shenyang City, Liaoning, China 110016

[*]Corresponding author: huishanw@126.com

Supplementary Tables

Supplementary Table S1: Primers used in quantitative real-time PCR

Gene name	Oligo name	Sequence (5'-3')
Kaiso	sense	CAGGGCAGTTATTAGGAGTGAAA
	antisense	CAGAATCAGGAGGTAAAGGCTCA
p120ctn	sense	TTCAGGCTGAGATTGGGCAG
	antisense	AGAACCACTCATCTTTGCCCT
BIK	sense	ACGACCAGACTGAGGACATCA
	antisense	CACCTGTTCGCAGGACACC
BAX	sense	AGAGGTCTTTTTCCGAGTGGC
	antisense	TGATGGTTCTGATCAGTTCCG
BCL2	sense	TCGTTGCCTTATGCATTTGTT
	antisense	ACTTGATTCTGGTGTTTCCCC
ACTB	sense	TCCTTCCTGGGCATGGAGT
	antisense	CAGGAGGAGCAATGATCTTGAT
GAPDH	sense	GGGTGTGAACCATGAGAAGTATG
	antisense	GATGGCATGGACTGTGGTCAT

Supplementary Table S2: Primers used in CHIP

Gene name	Primer pair	Oligo name	Sequence (5'-3')
BCL2	1	F(-3902)：	GTTTCCTTCTGGCTGCCCCC
		R(3601):	GATCCTCCTTCTTGCCCTTC
	2	F(-3602)：	GAAGGGCAAGAAGGAGGATC

		R(-3301):	CCAGAAAAGCACACCAAATT
	3	F(-3302):	AATTTGGTGTGCTTTTCTGG
		R(-3001):	GTGTGCAGTGGTGCGATCTC
	4	F(-3001):	GAGATCGCACCACTGCACAC
		R(-2700):	AAGCAGACAGTTTTAAATTA
	5	F(-2701):	TAATTTAAAACTGTCTGCTT
		R(-2400):	CCTAGTCCGTGGCCCAGGCC
	6	F(-2401):	GGCCTGGGCCACGGACTAGG
		R(-2100):	TGGGAGTGTGTGTGTCGCCT
	7	F(-2101):	AGGCGACACACACACTCCCA
		R(-1800):	GCGGCGCGGTGGGTGTGCGC
	8	F(-1801):	GCGCACACCCACCGCGCCGC
		R(-1500):	CTTACTTCATTCTCTGCACA
	9	F(-1501):	TGTGCAGAGAATGAAGTAAG
		R(-1200):	GCACCCCACCGGCGCACCCC
	10	F(-1201):	GGGGTGCGCCGGTGGGGTGC
		R(-900):	CGCTTCACGCCTCCCCAGGA
	11	F(-901):	TCCTGGGGAGGCGTGAAGCG
		R(-600):	CTCTCCAGTTATAGCTGATT
	12	F(-601):	AATCAGCTATAACTGGAGAG
		R(-300):	AAAACAAACTAATAAGTAAA
	13	F(-301):	TTTACTTATTAGTTTGTTTT
		R(-1):	GAGGAAAGGAGGAAAGTAAC
BAX	1	F(-971)	CCTGCTGATCTATCAGCACA
		R(-644)	GTGCAGTGGCCCAATCATGG
	2	F(-645)	CCATGATTGGGCCACTGCAC
		R(-317)	AGCTCTCCCCAGCGCAGAAG
	3	F(-316)	CTTCTGCGCTGGGGAGAGCT
		R(-1)	ACCGCCGCTCCCGCCGCCGC
BIK	1	F(-3001)	AAAATTAGCCGGGTGTGGTG
		R(-2700)	TTCAAGCTCCCCACCCTGCC
	2	F(-2701)	GGCAGGGTGGGGAGCTTGAA
		R(-2400)	GCTTTGTGCTCCTGGAGACC
	3	F(-2401)	GGTCTCCAGGAGCACAAAGC
		R(-2100)	CCCGCCTCTAATAAAAATAC
	4	F(-2101)	GTATTTTTATTAGAGGCGGG
		R(-1800)	GTTTGTTCACACAGGACACT
	5	F(-1801)	AGTGTCCTGTGTGAACAAAC
		R(-1500)	AAATCTAATGGCCTAGTTCA
	6	F(-1501)	TGAACTAGGCCATTAGATTT
		R(-1200)	CAGAGTGGGGAGGCAGAGAG
	7	F(-1201)	CTCTCTGCCTCCCCACTCTG
		R(-900)	TCACCCCATACAGTGTCTTA

	8	F(-901)	TAAGACACTGTATGGGGTGA
		R(-600)	AAGTTCTCCTCGGACACCAG
	9	F(-601)	CTGGTGTCCGAGGAGAACTT
		R(-300)	TGGAGGGCAGTGGCACAATC
	10	F(-301)	GATTGTGCCACTGCCCTCCA
		R(-1)	TTCTCCTCTGGGGCAAAAAG

Supplementary Table S3: Primers used in MSP

Gene name	Primer pair	Oligo name	Sequence (5'-3')
BAX	1	F(M)(-635)	TTTTAGTTTGGGTAATATAGTGAG
		R(M)(-516)	ATTACAAACATAAACTACCGCGC
		F(U)(-635)	TTTTAGTTTGGGTAATATAGTGAG
		R(U)(-516)	ATTACAAACATAAACTACCACAC
	2	F(M)(-532)	TAGTTTATGTTTGTAATTTTAGCGT
		R(M)(-428)	TAAATCTAACAATATAACCCACGCC
		F(U)(-532)	GTAGTTTATGTTTGTAATTTTAGTGT
		R(U)(-428)	TAAATCTAACAATATAACCCACACC
	3	F(M)(-404)	TTTAGTTTTTAGTTATTTATAACGTT
		R(M)(-302)	ATTTAAACTCTCCCCAACGCA
		F(U)(-404)	TTTAGTTTTTAGTTATTTATAACATT
		R(U)(-302)	ATTTAAACTCTCCCCAACACA

M:methylated, U: unmethylated

Supplementary Table S4: Primers for genomic DNA amplification in luciferase reporter assay

Gene name	Oligo name	Sequence (5'-3')
BCL2	F(MluI)	GATACGCGTGCTTCTAGCGCTCGGCACCG
	R(SmaI)	GATCCCGGGTGAGGAAAGGAGGAAAGTAA
BAX	F(KpnI)	GATGGTACCCCTGCTGATCTATCAGCACA
	R(XhoI)	GATCTCGAGACGTGAGAGCCCCGCTGAAC
BIK	F(MluI)	GATACGCGTGGTCAGCCACTGCAGCTCCA
	R(XhoI)	GATCTCGAGTCTGGGGCAAAAAGACAGCA

Underlined: inserted restrictive endonuclease recognition sites

Supplementary Table S5: Site-directed mutations in luciferase reporter assay

Gene name	Promoter position	Site mutation (5'-3')	Sequence similarity to 5'-TCCTGCNA-3'

BCL2	-1040 ～ -1033	TCCTGCCT → TCCAACCT	87.5%→ 62.5%
	-970 ～ -963	TCCTGCCT → TCCAACCT	87.5%→ 62.5%
	-728 ～ -721	TCCTGCGG → TCCAACGG	87.5%→ 62.5%
	-193 ～ -186	TCCTGCAT → TCCAACAT	87.5%→ 62.5%
BAX	-389 ～ -382	TCCTGCCT → TCCAACCT	87.5%→ 62.5%
BIK	-1070 ～ -1063	AGCTGCAA → AGAGGCAA	75% → 50%
	-695 ～ -688	CCCTGCAG → CGATGCAG	75% → 50%
	-390 ～ -383	CCCAGCTA → CGAAGCTA	75% → 50%
	-75 ～ -68	TCCTGTGA → TCCAATGA	87.5% → 62.5%
	-36 ～ -29	TCCAGTCA → TGAAGTCA	75% → 50%

Underlined: mutated nucleotides

Supplementary Table S6: Probes used in EMSA

Gene name	Oligo name	Sequence (5'-3')
BCL2	F(3709-WT)	AAATTTCCTGCATCTCATGC
	R(3709-WT)	GCATGAGATGCAGGAAATTT
	F(3709-MU)	AAATTTCCAACATCTCATGC
	R(3709-MU)	GCATGAGATGTTGGAAATTT
BAX	F(585-WT)	TAGGGGTCCAGTCATATGCT
	R(585-WT)	AGCATATGACTGGACCCCTA
	F(585-MU)	ATAACGTCCTGCCTGGAAGC
	R(585-MU)	GCTTCCAGGCAGGACGTTAT
BIK	F(-75-WT)	GGGTCATCCTGTGAGAGAGC
	R(-75-WT)	GCTCTCTCACAGGATGACCC
	F(-75-MU)	GGGTCATCCAATGAGAGAGC
	R(-75-MU)	GCTCTCTCATTGGATGACCC
	F(-36-WT)	TAGGGGTCCAGTCATATGCT
	R(-36-WT)	AGCATATGACTGGACCCCTA
	F(-36-MU)	TAGGGGTGAAGTCATATGCT
	R(-36-MU)	AGCATATGACTTCACCCCTA

Underlined: mutated nucleotides

Supplementary Figures

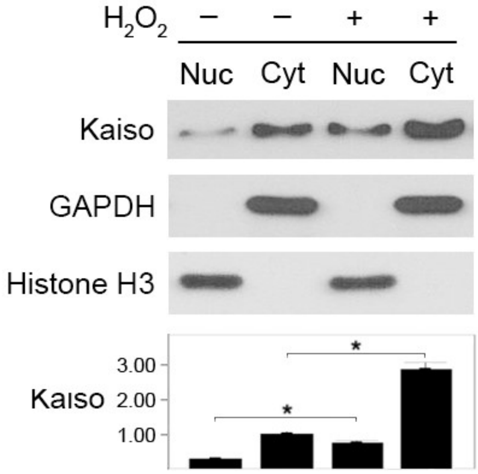

Supplementary Figure S1: Nuclear and cytoplasmic expression of Kaiso in H_2O_2 treated HMEC-1.

Nuclear and cytoplasmic extracts were separately prepared from HMEC-1 treated or untreated with 400μM H_2O_2 for 2 hours. The nuclear and cytoplasmic expressions of Kaiso were detected by Western blot. GAPDH served as a cytoplasmic marker and a loading control. Histone H3 served as a nuclear marker and a loading control. Values were presented as mean ± SD, * $p<0.01$, $n = 3$

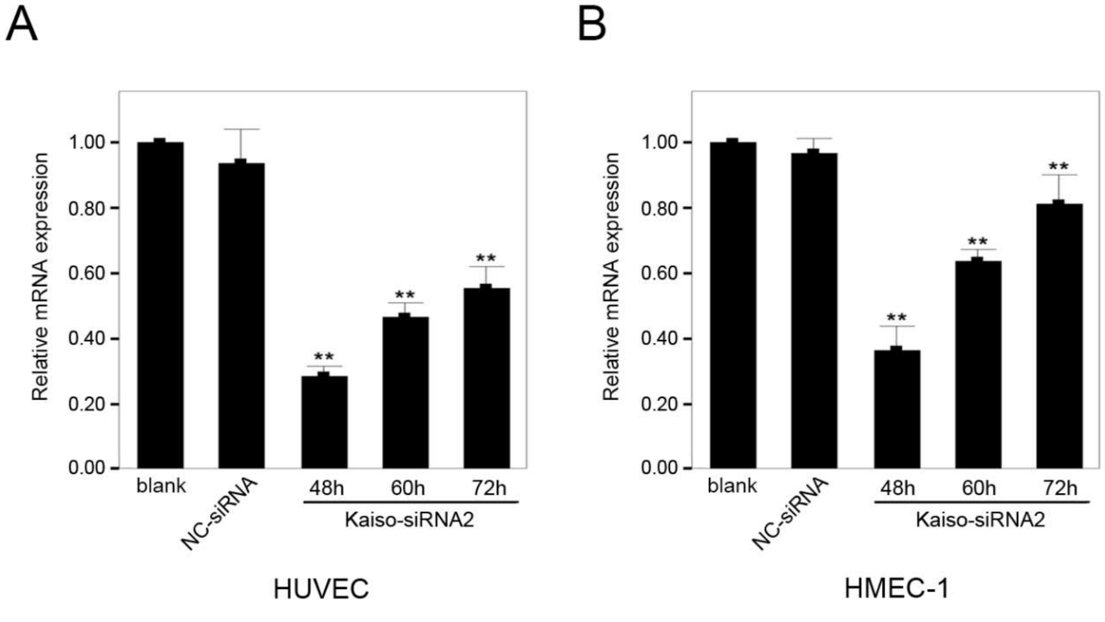

Supplementary Figure S2: Selective silencing of Kaiso in endothelial cells.

HUVEC (A) and HMEC-1 (B) were transfected with NC-siRNA or Kaiso-siRNA2 and silencing efficiency was evaluated by quantitative real-time PCR at 48, 60 or 72 hours post transfection. Values were presented as mean ± SD, * p<0.01, compared with blank, n = 3.

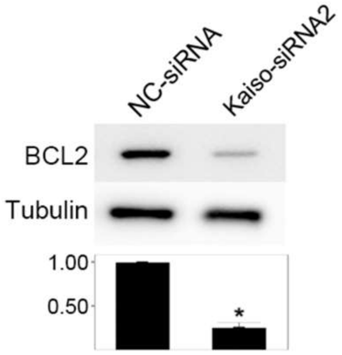

Supplementary Figure S3: Expression of BCL2 in Kaiso knock-down HUVEC.

HUVEC were transfected with NC-siRNA or Kaiso-siRNA2 and were cultured at 37°C for 48 hours. The expression of BCL2 was evaluated by western blot analysis. Tubulin served as a loading control. Values were presented as mean ± SD, * p<0.01, compared with blank, n = 3.

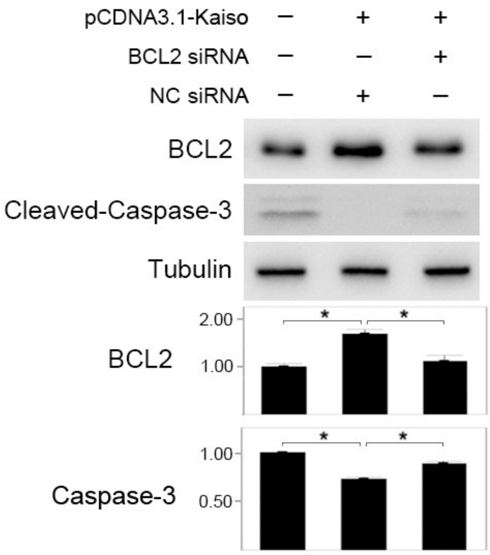

Supplementary Figure S4: Kaiso-mediated inhibition of Caspase-3 activation was compromised by BCL2 knock down.

HUVEC were cotransfected with pCDNA3.1-Kaiso and BCL2 siRNA.48 hours later, the cells were

treated with 400μM H_2O_2 for another 8 hours. The expressions of Kaiso, BCL2 and cleaved-Caspase3 were detected by Western blot. Tubulin served as a loading control. Values were presented as mean ± SD, * $p<0.01$, n = 3.

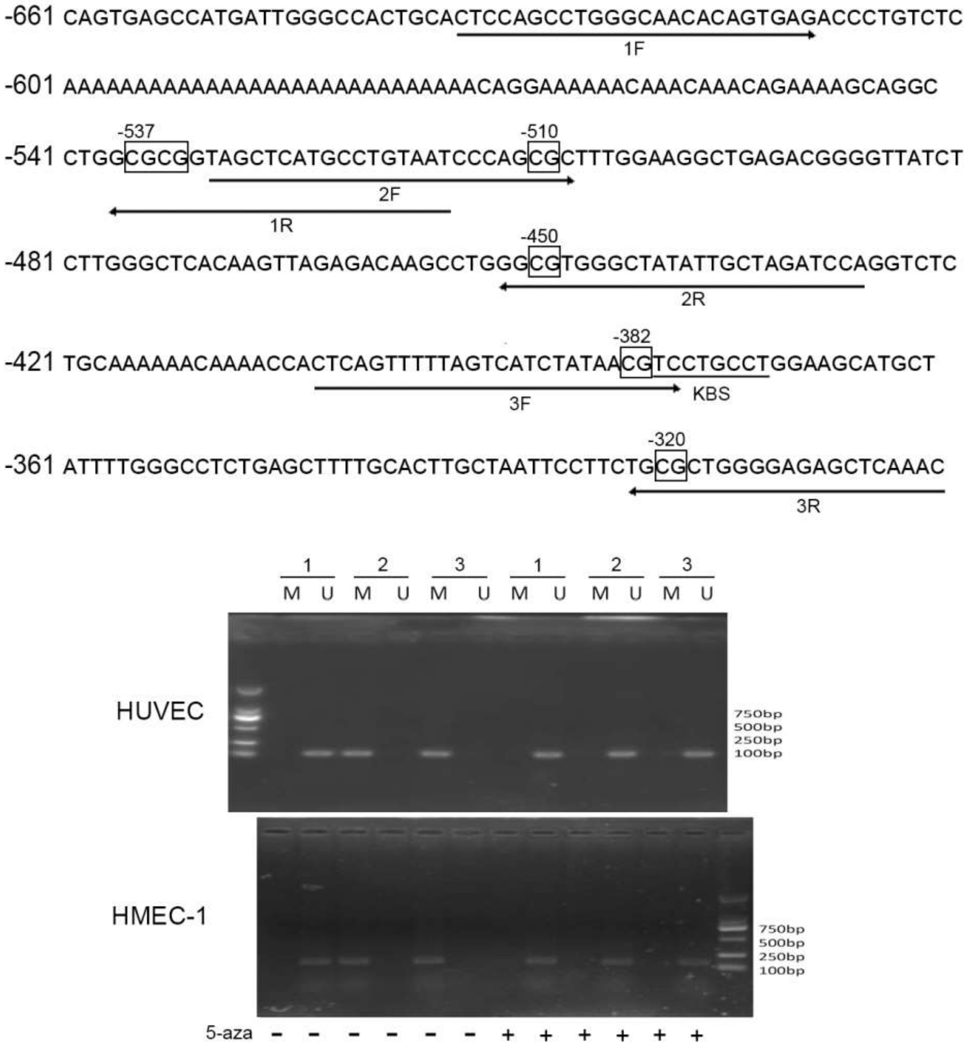

Supplementary Figure S5: Detection of ^{m}CpG in Kaiso binding region of the BAX promoter. Methylated cytosines were detected by methylation specific PCR (MSP) in HUVEC and HMEC-1 treated or untreated with 5-azacytidine for 72 hours. The PCR products were resolved in 3% agarose gel (lower). Three primer sets were used as shown in the upper panel (primers were listed in the Supplementary Table S3). CpGs detected were numbered and the KBS was underlined (upper). At least, four CpGs (-510, -450, -382, -320) around the KBS were methylated and all of which were demethylated after 5-azacytidine treatment. M: methylated primer pairs; U: unmethylated primer pairs.
